# Fluorine‐Free Biomass‐Derived Ionic Liquid Electrolytes: Ion Dynamics and Electrochemical Properties

**DOI:** 10.1002/chem.202501641

**Published:** 2025-08-20

**Authors:** Sayantika Bhakta, Gaurav Tatrari, Maiia Rudakova, Andrei Filippov, Faiz Ullah Shah

**Affiliations:** ^1^ Chemistry of Interfaces Luleå University of Technology Luleå SE‐971 87 Sweden

**Keywords:** fluorine‐free electrolytes, ionic interactions, ionic liquids, supercapacitors, transport properties

## Abstract

Here we present the synthesis, physical characterization, and transport as well as electrochemical properties of a novel class of ten ionic liquids (ILs) derived from biomass. Two biomass derived anions such as furan‐2‐carboxylate [FuA] and tetrahydrofuran‐2‐carboxylate [HFuA] are coupled to a range of nitrogen heterocyclic cations to create the ILs, for which the nature of cation controlled their properties. For instance, the thermal decomposition temperature ranges from 183 to 259 °C, the glass transition temperature from − 47 to − 70 °C, and the ionic conductivity from 0.002 to 1.4 mS cm^−1^ at 20 °C. The supercapacitors prepared using [EPy][FuA] and [EMPip][FuA] exhibited specific capacitances of 99 F g^−1^ and 70 F g^−1^ at 0.2 A g^−1^, respectively. The [EPy][FuA]‐based supercapacitor achieved an energy density of 56 Wh kg^−1^ as well as a power density of 410 W kg^−1^ at 0.2 A g^−1^, while the [EMPip][FuA]‐based supercapacitor achieved an energy density of 36 Wh kg^−1^ and a power density of 360 W kg^−1^ at 0.2 A g^−1^. In addition, the supercapacitors retained 98% and 94% of their initial capacitances after 6000 cycles, for [EPy][FuA] and [EMPip][FuA] electrolytes, respectively.

## Graphical abstract

This study presents the synthesis, physical characterization, and transport as well as electrochemical properties of a novel class of ten ionic liquids (ILs). The ILs are created with two biomass derived anions such as furan‐2‐carboxylate [FuA] and tetrahydrofuran‐2‐carboxylate [HFuA] coupled to a range of nitrogen heterocyclic cations, for which the nature of cation controlled their transport and electrochemical properties.

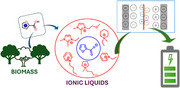

## 1 Introduction

Ionic liquids (ILs) are organic salts that are usually liquid at ambient temperature and possess a combination of properties including high thermal and electrochemical stabilities, nonflammability as well as structural designability, and are being looked at as a viable replacement for the volatile and toxic organic solvent‐based electrolytes widely used in energy storage devices (ESDs).^[^ ^1^, ^2^, ^3^, ^4^, ^5^, ^6^, ^7^ ^]^ Adoption of IL structure and a suitable combination of cation and anion might enhance the safety and performance of ESDs. This is primarily due to their quick ion transport and broad electrochemical stability windows (ESWs). Among cations, the tetraalkylammonium, and cyclic quaternary ammonium are the most widely studied cations for IL‐based electrolytes.^[^ ^8^, ^9^, ^10^ ^]^ These cations are generally coupled to the most popular fluorinated anions such as bis(trifluoromethylsulfonyl)imide (TFSI) and bis‐(fluorosulfonyl)imide (FSI) to create IL‐based electrolytes. However, the large amount of fluorine in these conventional ILs creates serious safety and environmental problems not only at the synthesis and implementation levels but also at the recycling stages. According to life cycle assessment (LCA), fluorinated anions are synthesized using hazardous compounds as precursors, mainly hydrogen fluoride gas (HF). The HF emissions, particularly during electric vehicle fire incidents and/or battery recycling processes, pose severe risks to both human health and the surrounding environment.^[^ ^11^, ^12^ ^]^

The development of efficient ESDs is critically important for our modern society and thus efforts are being made to design new functional materials for their optimum performance.^[^ ^13^, ^14^, ^15^, ^16^, ^17^ ^]^ Bearing in mind the sustainability issues, supercapacitors are highly desired due to their high capacitance, longer discharge currents, and outrageous cycle longevity.^[^ ^18^ ^]^ Supercapacitors are made and employed in a variety of applications where they can potentially replace traditional batteries either partially or fully.^[^ ^19^, ^20^ ^]^ Since most of the research is focused on the development of electrode materials for supercapacitors, there is a lack of functional electrolytes that can offer the required properties as well as safety. An electrolyte is a vital component of supercapacitors and responsible for the transfer and equilibrium of charges between the two electrodes. Despite possessing high capacitance and ionic conductivity, aqueous electrolyte‐based devices suffer from electrochemical instability, lower energy density, and unstable cycling performance. Unlike aqueous electrolytes, organic electrolytes provide relatively higher ESWs, but they are flammable and thermally unstable.^[^ ^21^, ^22^ ^]^ In this context, ILs can present all the unique properties that play a vital role in the performance of supercapacitors over wide temperature and potential ranges.

The combination of cations and anions strongly determines the overall properties of ILs. From the structural perspective of IL cations, *N*‐heterocycles such as pyrrolidinium (Pyrr), imidazolium (Im), pyridinium (Py), piperidinium (Pip) rings are very interesting synthons for the development of new task‐specific ILs due to their small compact sizes.^[^ ^23^, ^24^ ^]^ A stable positive charge is produced upon quaternization of the tertiary N‐atom, thereby ionizing the ring.^[^ ^25^ ^]^ However, many of the known ILs are based on fluorinated anions and their properties and applications are widely studied not only in energy storage but also in various other applications.^[^ ^21^, ^22^, ^26^, ^27^ ^]^ Again, the presence of fluorine and other halogens in ILs creates enormous problems, especially when used on industrial scales, and, therefore, the development of fluorine‐free and greener anions is inevitable for IL‐based electrolytes. We have recently introduced a number of new ammonium‐ and phosphonium‐based ILs coupled to fluorine‐free anions, furan‐2‐carboxylate [FuA], and tetrahydrofuran‐2‐carboxylate [HFuA].^[^ ^28^, ^29^, ^30^ ^]^ These ILs have the potential to improve the electrochemical properties as well as safety and sustainability issues of supercapacitors as they are derived from furoic acid, which is produced lignocellulosic biomass.^[^ ^31^, ^32^ ^]^

Motivated by the sustainability and enduring physical and electrochemical properties of the phosphonium‐ and ammonium‐based ILs with [FuA] and [HFuA] anions,^[^ ^30^ ^]^ we embarked on the development of new ILs based on various *N*‐heterocyclic cations. The reason for selecting *N*‐heterocyclic cations is their small and compact sizes, biodegradability and lower environmental impacts. Imidazolium‐ and pyridinium‐based ILs have been extensively used in medicine and pharmaceutical products as well as for dissolution of cellulose as greener solvents.^[^ ^33^, ^34^, ^35^ ^]^ Furthermore, Hernández‐Fernández et al. have demonstrated the low toxicity of imidazolium‐ and pyrrolidinium‐based ILs toward *Escherichia coli* for bioprocesses application.^[^ ^36^ ^]^ In this work, [FuA] and [HFuA] anions are utilized in the creation of ten different ILs for applications in supercapacitors. A thorough comprehension of the physicochemical and electrochemical traits of these novel ILs is presented and discussed in detail.

## 2 Experimental Section

### 2.1 Materials

Tetrahydro‐2‐furoic acid (99% purity; Sigma Aldrich), 2‐furoic acid (99% purity; Sigma Aldrich), 1‐methylpyrrolidine (99% purity; Sigma Aldrich), 1,2‐dimethylimidazole (99% purity; Sigma Aldrich), pyridine (99% purity; Merck), *N*‐methylpiperidine (99% purity; Acros organic), 1‐butyl bromide (99% purity; Sigma Aldrich), 1‐ethyl bromide (99% purity; Sigma Aldrich), and dichloromethane were used without any further purification. The description of synthesis and characterization of the ILs is provided in the Supporting Information (SI). All the prepared ILs were kept in a vacuum oven at 80 °C for about 7 days until the water content was measured below 100 ppm (Table 1), as determined by Karl Fischer titration using a 917 Coulometer (Metrohm) placed inside a glovebox with water and oxygen contents of less than 1 ppm.

**Table 1:** Thermal properties and ionic conductivities of the synthesized ILs.

Ionic liquid	*T*_g_ (°C)	*T*_d_ (°C)	σ at 20 °C (mS cm^−1^)	σ at 60 °C (mS cm^−1^)	Water content (ppm)
[BMPyrr][FuA]	−60	217	0.20	3.93	54 ± 5
[BMPyrr][HFuA]	−70	183	0.41	3.43	60 ± 5
[EMPyrr][FuA]	−69	239	0.50	4.54	61 ± 5
[EPy][FuA]	−66	213	1.41	9.91	60 ± 5
[BPy][FuA]	−54	230	0.23	2.77	56 ± 5
[EMPip][FuA]	−61	221	0.07	1.64	70 ± 5
[BMPip][FuA]	−54	227	0.02	0.61	62 ± 5
[EMMIm][FuA]	−51	253	0.11	2.22	61 ± 5
[BMMIm][FuA]	−47	251	0.002	1.26	63 ± 5
[BOMMIm][FuA]	−47	259	0.15	2.73	22 ± 5

### 2.2 Electrode Preparation

Multiwalled carbon nanotubes (MWCNTs) purchased from Sigma Aldrich (50–90 nm diameter) were used as the electrode material for supercapacitors (SCs). First, the binder solution was made by dissolving 20 mg of polyvinylidene fluoride (PVDF) in 20 ml of *N*‐methyl‐2‐pyrrolidone (NMP) and stirred overnight at 40 °C. Then 20 mg of the activated carbon purchased from nanografi (nano‐powder size < 100 nm) was added to 160 mg of MWCNT and ground for 30 minutes using a mortar grinder to create an even‐sized powder. The binder solution was then added dropwise to the powder while continuously mixing with a mortar grinder until a homogenous slurry was formed. Finally, the battery‐grade aluminum foil was properly cleansed and dried in a vacuum oven for about one hour, followed by coating using a doctor's blade technique to equally spread the slurry with 150 mm thickness over the aluminum foil. The prepared electrodes were placed in a vacuum oven at 90 °C for two days before being cut into 14 mm electrodes. Finally, the supercapacitor devices were made using two identical electrodes separated by an electrolyte‐soaked separator (Whatman filter paper, G/FD grade), which was placed under vacuum at room temperature before the device preparation to improve electrolyte absorption into the pores of the separator. The electrolyte‐soaked separator was then placed between the two electrodes, and the CR 2032‐coin cells were created.

### 2.3 NMR Spectroscopy and Mass Spectrometry

The structure and purity of the synthesized products were analyzed using nuclear magnetic resonance (NMR) and electrospray ionization mass spectrometry (ESI‐MS). NMR measurements were performed on Bruker Ascend Aeon WB 400 (Bruker BioSpin AG, Fällanden, Switzerland) NMR spectrometer. CDCl_3_ was used as solvent, and the working frequencies were 400.21 MHz for ^1^H and 100.64 MHz for ^13^C. Data were processed using Bruker Topspin 3.5 software. The ^1^H NMR spectra of the neat ILs as a function of temperature were measured by taking the sample in a 5 mm standard NMR tube.

For mass spectrometry, the samples were dissolved in acetonitrile and run on a Sciex Pulsar QTOF instrument using direct introduction via syringe pump at 150 microliter/min. The instrument scanned one scan per second from 50 to 800 Da and an external mass calibration was performed.

### 2.4 Thermal Analysis

Thermogravimetric analysis (TGA) was conducted using a PerkinElmer TGA 8000 instrument under nitrogen (N_2_) atmosphere using a heating rate of 10 °C per minute. Approximately 2–4 mg of the sample was utilized for each experiment. The onset of decomposition temperature (*T‐onset*) was computed by identifying the intersection point between the baseline weight and the tangent of the weight‐versus‐temperature curve, employing the Pyris software. Differential scanning calorimetry (DSC) was carried out with a PerkinElmer DSC 6000, using 2–5 mg of the sample placed in an aluminum pan. Both cooling and heating traces were recorded at a scanning rate of 5 °C per minute. To create an inert environment within the sample chamber, dry N_2_ gas was consistently supplied at a flow rate of 20 mL per minute. The glass transition temperature (*T_g_*) was determined by analyzing the inflection mid‐point of the initial S‐shaped transition slope using the Pyris software.

### 2.5 Ionic Conductivity

The ionic conductivity was assessed through impedance measurements in a frequency range from 1 Hz to 1 MHz, using an AC voltage amplitude of 10 mV. All the measurements were conducted via heating and cooling over a temperature range from − 20 to 100 ± 0.1 °C. A two‐electrode configuration was utilized, featuring a glassy carbon as the working electrode (WE) and a 70 µL platinum (Pt) crucible as both the sample container and the counter electrode (CE). The cell was thermally equilibrated for 10 minutes prior to each impedance measurement. The electrodes were polished with 0.25 µm Kemet diamond paste before each experiment, and the cell constant was calculated using a Metrohm 100 µS cm^−1^ KCl standard solution (Kcell = 1.8736 cm^−1^).

The relationship between temperature (*T*) and ion conductivity (σ) was fitted using the Vogel–Fulcher–Tammann (VFT) equation (Equation 1), where σ_0_ represents a pre‐exponential factor, B and *T*_0_ are adjustable parameters and R is gas constant. B is linked with activation energy (*E_σ_*) of the system where *E_σ =_* B × R, while the *T*_0_ is associated with the ideal vitreous transition temperature, representing the point where configurational entropy vanishes.

(1) $$\sigma =\sigma_{0}\;\;exp\frac{-B}{R\left(T-T_{0}\right)}$$

### 2.6 NMR Diffusometry

Pulsed‐field gradient (PFG) NMR diffusometry was conducted using a Bruker Ascend Aeon WB 400 (*Bruker BioSpin AG*) nuclear magnetic resonance (NMR) spectrometer with the working frequencies were 400.21 MHz for ^1^H, using a Diff50 PFG NMR probe (Bruker). The maximum magnetic field gradient pulse amplitude was 29.73 T m^−1^. Samples were positioned within a standard 5 mm NMR glass tube and before each measurement, the sample was allowed to stabilize at a specific temperature at least for 20 minutes.

The diffusivity of a molecule is quantified by the diffusion decay (DD) of the amplitude (A) of the NMR spectral line, derived through Fourier transformation of a descending half of the stimulated echo (StE). This decay, as a function of the amplitude of the applied pulsed field gradient, can be expressed by Equation 2 for a simple nonassociating molecular liquid under the employed stimulated echo pulse sequence:^[^ ^37^, ^38^ ^]^

(2) $$A\;\left(g,\delta ,t_{d}\right)=A\left(0\right)\exp\left(-\gamma^{2}g^{2}\delta^{2}Dt_{d}\right)$$

Here, A(0) represents the factor proportional to the proton content in the system, as well as the spin‐lattice and spin‐spin relaxation times. A is the integral intensity of the NMR signal, τ and τ_1_ are the time intervals in the pulse train; *g* is the gyromagnetic ratio for magnetic nuclei; g and δ are the amplitude and the duration of the gradient pulse; t_d_ = (Δ – $\frac{\delta }{3}$) is the diffusion time; Δ = (τ + τ_1_). D is the diffusion coefficient. In the measurements, δ was in the range of (0.5 – 3) ms, τ was in the range of (3 – 5) ms, and *g* was varied from 0.06 to 29.73 T m^−1^. Diffusion time t_d_ varied from 20 to 100 ms. The recycle delay during the accumulation of signal transients was 5 s.

The diffusivity data is subjected to analysis by fitting the data into the following VFT Equation 3:

(3) $$D=D_{0}\;\;exp\frac{-B}{R\left(T-T_{0}\right)}$$

The adjustable parameters, D_0_, *T*_0_, and B, are involved in determining the energy of activation for diffusion, where the activation energy (E_D_) is correlated with B as E_D_ = B × R. We characterized the temperature‐dependent diffusion coefficient, D(*T*), by fitting the parameters D_0_, T_0_, and B, while R is a gas constant.

### 2.7 FTIR Spectroscopy

For Fourier transform infrared (FTIR) spectroscopy, attenuated total reflection (ATR‐FTIR) spectra were captured using a Bruker IFS 80v spectrometer. The instrument had a deuterated triglycine sulfate (DTGS) detector and a diamond ATR accessory, operating in the double‐side forward‐backward acquisition mode. The spectra were obtained with a total of 64 scans, co‐added, and signal‐averaged, at an optical resolution of 4 cm^−1^.

### 2.8 Electrochemical Assessments

The coin cells were tested using a Biologic BCS‐810 battery testing instrument. Initially, the cyclic voltammetry (CV) was performed at 50 mVs^−1^ with a potential window of 2 V to test the feasibility, stability, and reversibility. The CV was performed at different scan rates from 1 to 100 mV s^−1^ at two different temperatures, 30 and 60 °C. Electrochemical impedance spectroscopy (EIS) was performed in the frequency range from 0.01 to 10^6^ Hz, and the charge‐discharge was carried out at different current densities. All the electrochemical experiments were performed using a closed cell using a temperature‐controlled climate chamber. The specific capacitance (Cs) was calculated from CV and GCD using Equations 4 and 5, respectively.^[^ ^25^, ^39^ ^]^

(4) $$\mathrm{Cs}\;=\frac{\int idV}{2mK\Delta V}\;$$

(5) $$Cs=\frac{I\Delta t}{m\Delta V}\;$$

where C_s_ is the specific capacitance in F g^−1^, idv is the area under the CV curve, m is the mass in mg (1.5 mg each electrode), K is the scan rate in mV s^−1^, ΔV is the potential window in V, I is current in mA, and Δt is discharge time. Finally, the energy density (E_D_) and power density (P_D_) were evaluated using Equations 6 and 7, respectively.^[^ ^24^, ^39^, ^40^, ^41^ ^]^

(6) $$\mathrm{Energy}\;\mathrm{density}\;=\frac{CV^{2}}{3\cdot 6\times 2}\;$$

(7) $$\mathrm{Power}\;\mathrm{density}\;=\frac{\mathrm{Energy}\;\mathrm{density}\;X\;3600\;}{\Delta t}\;$$

## 3 Results and Discussion

We begin with a brief description of the synthesis and structural characterization of the synthesized ILs, followed by discussion of their thermal stabilities and phase transitions. Subsequently, the transport properties such as ionic conductivity and ion diffusion of all the ILs are described as a function of temperature together with the possible interactions between the cations and anions using FTIR and NMR spectroscopic techniques. Lastly, the application of these ILs as electrolytes in symmetric supercapacitors is thoroughly presented and discussed.

### 3.1 Synthesis and Characterization

**Figure 1:**
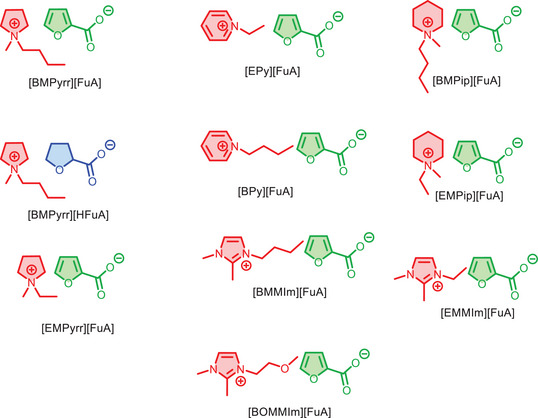
Structures and abbreviations of the ionic components of the synthesized ILs.

Synthesis of the ten ILs is carried out using several synthetic protocols. First, the cations are prepared through alkylation of the *N*‐heterocyclic rings using alkyl bromides. In the second step, the bromide salts are converted to hydroxide salts using silver oxide. Finally, the ILs are synthesized via a simple neutralization reaction between either 2‐furoic acid or tetrahydro‐furoic acids and the hydroxide salts of the cation. The final products were extracted with dichloromethane and dried over sodium sulphate to remove the traces of water. All the ILs are obtained in quantitative yields, > 90%. The synthesized ILs are designed in such a way as to investigate the effect of the structure of the cations, the length of alkyl chains on the cations, and the aromaticity of cations and anions. The chemical structures and abbreviations of all the synthesized ILs are shown in Figure 1.

The ESI‐MS (Figures S1‐S10) and ^1^H, ^13^C NMR (Figures S11‐S30) spectra confirmed the structures and purity of the final products. ^1^H NMR reveals the neutral furoic acid to exhibit three distinct ^1^H resonance lines for aromatic groups at 7.5 (singlet), 6.9 (multiplet), and 6.5 (multiplet) and the ^1^H resonance line for the acidic proton at 8.54 − 10.00 ppm. The ^1^H resonance lines for the aromatic groups slightly shifting upfield, while the resonance line for the acidic proton disappeared entirely upon conversion to the furoate anion – confirms the effective synthesis and purity of the synthesized ILs. Furthermore, the ^1^H resonance lines for aromatic groups for the furoate anion shifted slightly upfield upon altering the alkyl chain length of the cations, likely due to changes in ionic interactions between cation and anion, and ion orientation.^[^ ^42^ ^]^ Similarly the ^1^H resonance lines for furoate anion shifted upfield with changing the cation from [BMPyrr] to [BMMIm].

The aromaticity of the anion has also influenced the environment of the cation, as changing from the aromatic [FuA] to nonaromatic [HFuA] resulted in a slight downfield shift of the resonance lines for [BMPyrr] cation. As compared to [FuA] anion, the protons in the nonaromatic [HFuA] anion exhibited a different ^1^H resonance pattern due to its chiral conformation and saturated structure. Additionally, introducing an ether group into the alkyl chain of the imidazolium cation causes the cation and anion resonance lines to shift toward the deshielding zone, clearly illustrating the influence of functional groups on the structure of the ILs. Comparing the aromatic pyridinium cation with the pyrrolidinium cation, the ^1^H resonance lines of the aromatic [FuA] anion are shifted upfield, attributed to the delocalization of the positive charge over the aromatic rings. All the characteristic changes are observed in the ^1^ ^3^C NMR spectra of these ILs as well.

### 3.2 Thermal Analysis

**Figure 2:**
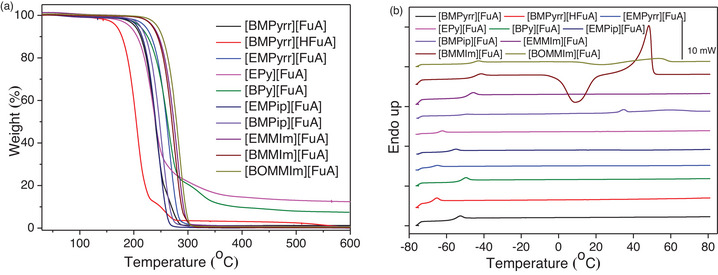
a) TGA thermograms and b) DSC curves of the synthesized ILs.

The dynamic TGA data reveal the decomposition temperatures (*T*_d_) of all the ILs to be in the range from 180 to 260 °C (Figure 2a), which is in accordance with the previous reports on FuA and HFuA based ILs comprising ammonium and phosphonium cations.^[^ ^29^, ^31^ ^]^ The imidazolium‐based ILs exhibit relatively higher thermal stabilities with single‐step decompositions as compared with the structural analogous pyrrolidinium‐, pyridinium‐, and piperidinium‐based ILs. The decomposition of butyl‐functionalized pyrrolidinium‐ and pyridinium‐based ILs occurred in two steps with about 70% weight loss in the first step. With a common anion, the [BOMMIm][FuA] IL featuring ether chain reveals a higher decomposition temperature than the [BMMIm][FuA] IL without ether moiety, clearly indicating the repulsive force between the electron‐withdrawing effect of the oxygen atom from the alkyl chain and methyl group of the imidazolium ring on thermal stability.^[^ ^8^, ^43^, ^44^ ^]^

Apart from this, having high molecular weight and higher intermolecular attractions also lead to higher decomposition temperatures.^[^ ^10^ ^]^ As expected, the [BMPyrr][HFuA] IL with a nonaromatic anion exhibits the least decomposition temperature, and agrees well with the previous study.^[^ ^28^ ^]^ Overall, the thermal stabilities of these fluorine‐free ILs are higher than the oligoether‐based ILs,^[^ ^45^ ^]^ comparable with phosphate based ILs^[^ ^24^, ^46^ ^]^ and slightly lower than NTf_2_, PF_6_ and BF_4_‐based ILs.^[^ ^8^, ^10^ ^]^ However, a similar study with Pyrr, Pip, and Im‐based fluorinated ILs described that the use of TFSI, FSI, and OTf anion increases the thermal stability of corresponding alkyl‐and ether‐functionalized ILs.^[^ ^44^, ^47^, ^48^, ^49^, ^50^, ^51^ ^]^

All the ILs are classified as glass‐forming liquids, as they exhibit glass transition temperatures (*T*_g_) ranging from − 47 to − 70 °C (Figure 2b). The Pyrrolidinium‐ and Pyridinium‐based ILs have lower *T*_g_ values than imidazolium‐based ones, indicating weaker cation‐anion interactions. The pyrrolidinium‐based IL with butyl chain shows lower *T*_g_ than the corresponding IL with ethyl chain, however, it is the opposite in the case of pyridinium‐, and piperidinium‐, and imidazolium‐based ILs. The imidazolium‐based ILs showed comparatively higher *T*_g_ values, probably due to the aromatic ring facilitating π‐π stacking and restricting the free rotation of the alkyl chain and ether chains.^[^ ^51^ ^]^ The [BMMIm][FuA] exhibits supercooling behavior and the glass transition is followed by − 47 °C. Furthermore, the [BMPyrr][HFuA] IL with a nonaromatic anion has the lowest *T*_g_ of − 70 °C, indicating weaker interactions between [BMPyrr] cation and [HFuA] anion. Compared to ILs with other anions, these ILs exhibit relatively higher Tg values than diisocyanate‐based ILs and are comparable to or sometimes lower than those of TFSI‐, FSI‐, and BETI‐based ILs.^[^ ^8^, ^43^, ^52^ ^]^

### 3.3 Transport Properties

**Figure 3:**
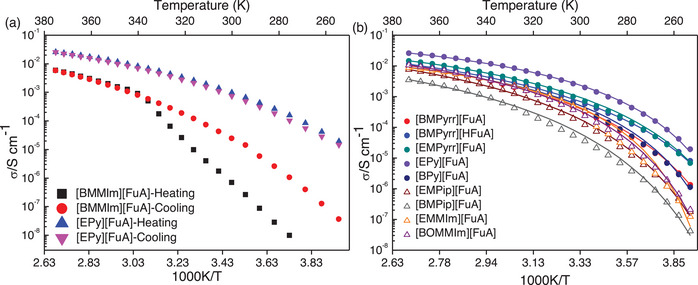
Ionic conductivity as a function of temperature, a) heating and cooling cycles of [BMMIm][FuA] and [EPy][FuA], and b) heating cycles of the other ILs. The solid lines illustrate the correlation of the data using the Vogel‐Fulcher‐Tamman (VFT) equation.

Ionic conductivities of all the ILs are measured during heating and cooling cycles, and the values perfectly match for all the ILs except for [BMMIm][FuA] (Figure 3a). This special behavior of [BMMIm][FuA] could be due to supercooled behavior, which has already been observed in the DSC analysis. Likely due to decreasing ionic interactions, the ionic conductivity of all the ILs increases with increasing temperature and [EPy][FuA] IL exhibits the highest ionic conductivity over the whole measured temperature range. This is attributed to the symmetric structure of the pyridinium cation, tends to be more mobile because they distribute their charge evenly, reducing ion pairing and the reduced cation‐anion interactions in [EPy][FuA]. However, this IL provides slightly lower ionic conductivity than the reported pyridinium‐based ILs with fluorinated anions.^[^ ^53^, ^54^ ^]^ The absence of aromaticity in [HFuA] anion resulted in higher ionic conductivity of [BMPyrr][HFuA] IL at lower temperatures than the aromatic [BMPyrr][FuA] IL, while the latter provides higher conductivity at higher temperatures indicating weaker ionic interactions with increasing temperature (Figure 3b).

Having a common [FuA] anion, the ILs functionalized with ethyl group reveal higher ionic conductivities compared to the ILs with butyl groups, suggesting the role of cation size in ionic mobility and agrees well the Stokes’ law. For instance, [EMPyrr][FuA] has an ionic conductivity of 0.50 mS cm^−1^ at 20 °C, while [BMPyrr][FuA] demonstrated 0.20 mS cm^−1^ at the same temperature. For the same anion, increasing the size of the cation from pyrrolidinium to piperidinium, reduces the the ionic conductivity. Parallel to the size of the cation, changing functional groups can also affect the ionic conductivity. Since the ether group in the alkyl chain of the imidazolium cation makes the IL more flexible, it results in higher ionic conductivity for [BOMMIm][FuA] than [BMMIm][FuA].^[^ ^43^ ^]^ The synthesized ILs exhibit higher conductivity than the previously reported fluorine‐free ILs containing bulkier anions,^[^ ^28^, ^30^, ^46^, ^55^, ^56^ ^]^ but lower compared to some fluorine‐free ILs based on selenocyanate (SeCN−), tricyanomethanide, and related anions.^[^ ^57^, ^58^ ^]^

The ion‐ion interactions decrease with increasing temperature leading to lower resistivity as observed in the EIS data (Figure S31). The ionic interactions are also clearly governed by the degree and balance of attractive forces. For imidazolium‐based ILs with varying alkyl chain lengths, this can be explained by van der Waals forces. Generally, ILs with different types of anions and cations but the same counterparts are influenced by the anionic Lewis basicity and the cationic Lewis acidity.^[^ ^59^ ^]^ The introduction of an additional nitrogen atom in the cation increases the polarity and thus leading to a decrease in the ionic conductivity of the IL. This trend is further supported by the higher glass‐transition temperature observed in the DSC data of the imidazolium cation‐based ILs. The VFT parameters (Table S1) show that the *E*_σ_ for Pyridinium‐based ILs are the smallest and highest for Piperidinium‐based ILs, which agrees with the ionic conductivity and DSC data and again indicates that higher thermal energy is required to reach the same ion mobility. Furthermore, the experimental *T_g_* obtained from the DSC are higher than the *T*_o_ and the *T_g _*− *T*_o_ are less than 50 K.

**Figure 4:**
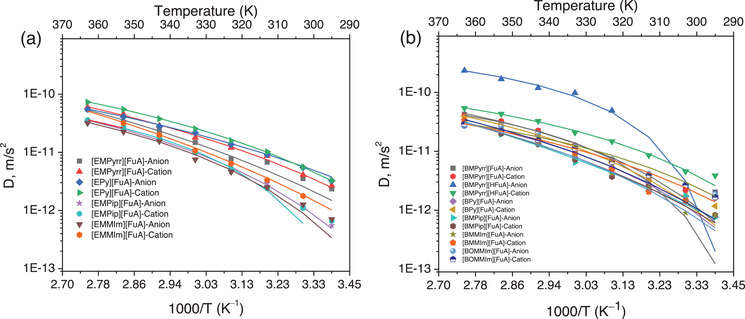
Diffusion coefficients of the ILs with ethyl a) and butyl‐chain b) substituted cations and anions. The symbols and solid lines are defined as the experimental points and the best‐fitting curve of the diffusion data to the VFT equation, respectively.

To further get a deeper insight into the individual ion mobility, PFG NMR diffusometry is employed and the data suggest that diffusivity of all ions increases as a function of temperature and follow the VFT functional behavior (Figure 4). This temperature‐dependent increase in ionic conductivity is associated with the thermal activation of the diffusion process.^[^ ^46^ ^]^ In the case of the [EPy][FuA] IL, both ions exhibit higher diffusivity compared to the other ILs, suggesting that ion–ion interactions have a significant influence rather than the ion size. The [EPy] cation and the [FuA] anion are stabilized by the enhanced charge delocalization or resonance effects associated with the carboxyl group in the anion. The other ILs containing ethyl groups follow the traditional rule, where the diffusivity of both cations and anions is correlated with their respective ion sizes.

Interestingly, the cations and anions in the [EMMIm][FuA] IL diffuse much faster than in the [BMMIm][FuA] IL, which contrasts sharply with the behavior observed in Pyrr‐based ILs. At elevated temperatures, the nonaromatic [HFuA] anion exhibits the highest diffusivity and different behavior with increasing temperature, likely due to the reduced intermolecular interactions with neighboring ionic species compared to other ILs.^[^ ^28^ ^]^ Overall, the D₀ value is higher for Pyrr‐based ILs than for Im‐based ILs, indicating a significant difference in ion mobility, primarily due to the smaller cation sizes and presumably weaker ion–ion interactions. Notably, the [BOMMIm][FuA] IL displays higher ion diffusivity than the Im‐based ILs, which can be attributed to the mesomeric effect of the methoxy group in the alkyl chain. Conversely, the [BMPip][FuA] IL exhibits the lowest anion diffusivity at higher temperatures, indicating stronger cation–anion interactions. The VFT parameters for ion diffusivity data (Table S2) including the *E*_σ_ agree well the ionic conductivity and ion diffusivity data.

### 3.4 Ionic Interactions

**Figure 5:**
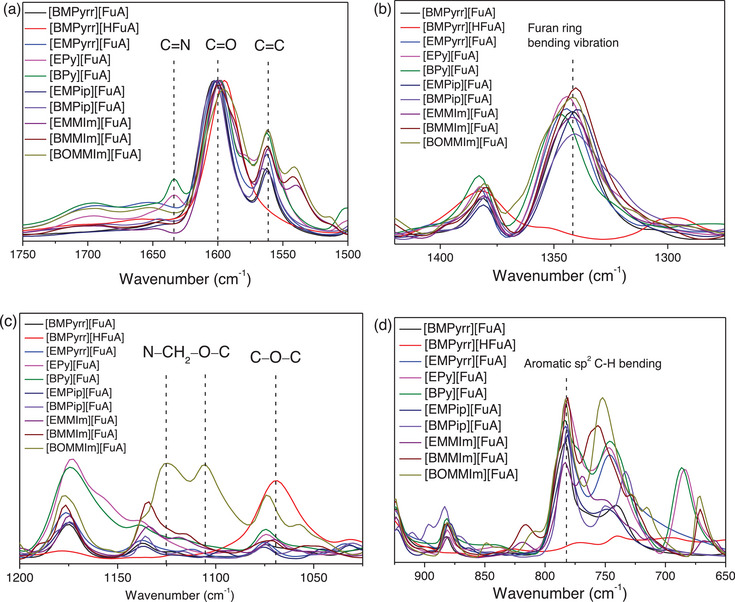
FTIR spectra (a, b, c, and d) of the synthesized ILs.

The ionic interactions in the neat ILs are analyzed using AT‐FTIR and ^1^H NMR spectroscopic techniques. FTIR spectra revealed the key vibrational features to understand the interactions between cations and anions in these ILs (Figures 5a–5d). The stretching band at 1633 cm^−1^, observed only in Py‐based ILs ([Epy][FuA] and [BPy][FuA]), corresponds to the C = N stretching in these ILs. The strong band at 1600 cm^−1^, attributed to the carboxylate (−COO^−^) stretching vibration of [FuA]^−^ anion in [BMPyrr][HFuA], shifts to lower wavenumbers when [FuA]^−^ is coupled to other cations, indicating enhanced electron delocalization and partial single‐bond character (Figure 5a). In contrast, the [P_4444_][FuA] IL exhibits a C = O stretching frequency around 1700 cm^−1^,^[^ ^28^, ^29^ ^]^ reflecting a pronounced double‐bond character. The C═C stretching vibrations in [FuA]^−^ anion appear around 1560 cm^−1^, and as expected, remained unaffected by changing the nature of cations. This vibration is absent in [BMPyrr][HFuA] IL and confirm the nonaromatic nature of the [HFuA] anion.

The ether functionalities are evident in the 1150–1050 cm^−1^ region (Figure 5c). The sharp peak at 1070 cm^−1^ in [BMPyrr][HFuA] corresponds to the symmetric stretching of the HFuA ring. Conversely, in [BOMMIm][FuA], the asymmetric ether chain induces a band at 1125 cm^−1^ with shoulders at 1105 and 1073 cm^−1^, reflecting interactions with the cation. The bending vibrations observed at 1477–1420, 1385–1342, and 781 cm^−1^ are attributed to the C─H, C = C─H, and aromatic sp^2^ C‐H bending groups, respectively.^[^ ^60^ ^]^ These vibrations are absent in the [BMPyrr][HFuA] IL. Altogether, the particular the shift of the carbonyl band to 1600 cm^−1^ along with the absence of O─H stretching, underscores the distinction between carbonyl and carboxylate groups and is consistent with the electron delocalization effects.

**Figure 6:**
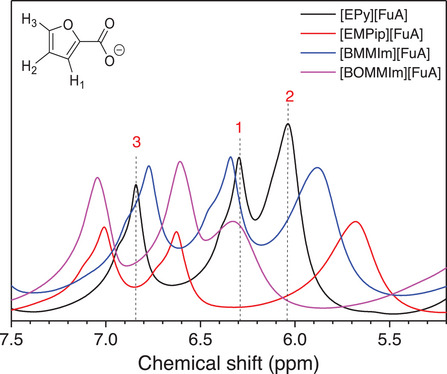
^1^H NMR spectra of the neat ILs with different cations at room temperature.

**Figure 7:**
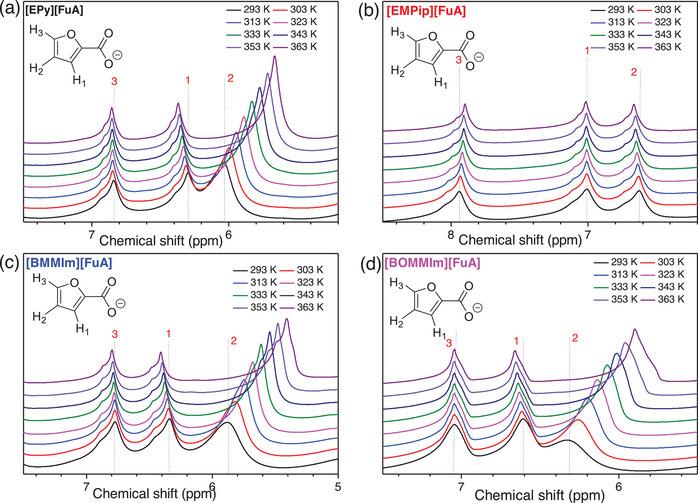
^1^H NMR spectra of the neat ILs (a–d) as a function of temperature.

To better understand the ions' local environment, several neat ILs are selected and variable temperature ^1^H spectroscopy is employed as a function of cation and temperature (Figures 6 and 7). In the case of [EPy][FuA], the ^1^H resonance lines at 6.84 (H3), 6.23 (H1), and 6.03 (H2) ppm of the aromatic furoate anion are shifted by changing the nature of cation, which is attributed to the different nature of interactions between the cation and aromatic anion – also clear from the ionic conductivity data. For [EMPip][FuA] IL, the H1 is shifted upfield while the H2 and H3 are shifted downfield, however, all the three resonance lines are shifted downfield in the case of [BOMMIm][FuA], suggesting that the protons are deshielded due to the presence of the ether chains in the cation. It means that the electron‐donating effect of the ether group potentially increases the electron density of the imidazolium ring and thus resulting in weaker interactions between cation and anion.^[^ ^61^ ^]^ The ^1^H resonance lines correspond to H1 and H3 display shoulders to the left indicating more than one mode of interactions of the [FuA] anion with the cation.

Clear evidence of temperature‐dependent ionic interactions between cation and anion is observed by variable temperature ^1^H NMR spectroscopy (Figure 7). The presence of broad ^1^H resonance lines in neat ILs compared to samples dissolved in CDCl_3_ indicates insufficient averaging of dipole‐dipole spin interactions in these systems due to their higher viscosities. A significant systematic shift in the ^1^H NMR resonance lines of the furoate anion has been observed with increasing temperature.^[^ ^46^ ^]^ This is primarily due to the weakening of ionic interactions and faster ion mobility at higher temperatures, as seen in the ionic conductivity and ion diffusion data. In the case of [EPy][FuA], resonance line for H2 is shifted upfield, while the lines for H1 and H3 are shifted downfield with increasing temperature and suggesting deshielding of the latter two protons (Figure 7a). For [EMPip][FuA], the ^1^H resonance line associated with H2 is shifted downfield, the line for H3 is shifted slightly upfield, whereas the resonance line for H1 remains unaffected with increasing temperature (Figure 7b).

In contrast to the pyridinium‐ and piperidinium‐based ILs, the imidazolium‐based ILs revealed different behavior with increasing temperature. For both [BMMIm][FuA] and [BOMMIm][FuA] ILs, the H3 remained less affected, H1 is shifted downfield, while the H2 is shifted significantly upfield as a function of temperature (Figures 7c and 7d). This shift indicates changes in the strength of hydrogen bonding between the methyl proton in the N–C(Me) = N– group and the oxygen atoms of the carbonyl group and furan ring, as well as decrease in the π‐π interactions between the aromatic imidazolium cations and the furoate anions. Additionally, the ether group in the alkyl chain of the [BOMMIm] cation diminishes the chance of cation‐anion hydrogen bonding by promoting self‐hydrogen bonding due to its electron‐donating nature and the free rotation of the alkyl chain.^[^ ^44^ ^]^ Altogether, unlike [EMPip][FuA], the H1 in the ILs with both aromatic cations and anions such as [EPy][FuA], [BMMIm][FuA], and [BOMMIm][FuA] is shifted to downfield with increasing temperature, which is due to the additional π‐π interactions in these ILs.

### 3.5 Electrochemical Assessments

**Figure 8:**
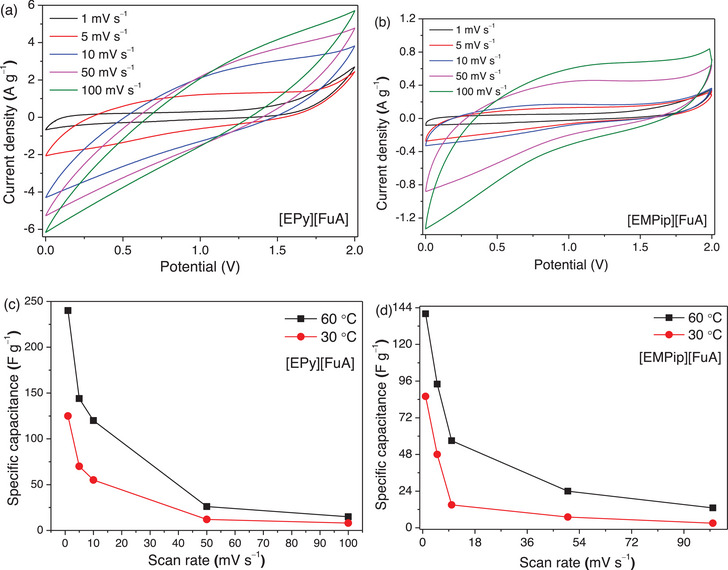
Cyclic voltammograms using different scan rates at 60 °C for SCs with [EPy][FuA] IL a) and [EMPip][FuA] IL b) as electrolytes. Specific capacitance as a function of temperature and scan rates at 30 °C and 60 °C for [EPy][FuA] c) and [EMPip][FuA] d) electrolytes.

The electrochemical performance of the [EPy][FuA] and [EMPip][FuA] ILs is evaluated as electrolytes in MWCNT‐based symmetric SCs. The reason for choosing MWCNTs as electrode materials is their commercial availability, well understood structures and properties, as well as structural flexibility and good electrochemical properties. The aromatic [EPy][FuA] IL electrolyte displays a much better performance compared to its structural analogous [EMPip][FuA] IL (Figure 8), which is attributed to its higher ionic conductivity and ion diffusivity.^[^ ^39^ ^]^ As expected, the specific capacitance decreases with increasing scan rate, however. it increases as a function of temperature (Figures 8a–d and S34), where the latter is enabled by higher thermal stability, improved ion–ion dissociation, and faster ion mobility at elevated temperatures.^[^ ^24^ ^]^

The overall reversibility of the oxidation and reduction halves of the CVs is improved for both electrolytes by increasing the temperature from 30 °C to 60 °C, demonstrating stable CV performance and better electrochemical stability at higher temperatures (Figures 8a,b, and Figure S32). The [EPy][FuA] IL‐based SC shows typical capacitive behavior, with a uniform current–potential fluctuation, while a relatively modest distortion and poor capacitive performance are observed for the [EMPip][FuA] IL‐based SC.^[^ ^24^, ^40^ ^]^ The faster ion mobility of [EPy][FuA] IL electrolyte results in a better contact between the electrolyte and the electrode surface, leading to a better capacitance performance as compared to [EMPip][FuA].^[^ ^39^, ^40^ ^]^ In addition, the variable voltage CVs at scan rates of 50 mV s^−1^ and 100 mV s^−1^ show that the [EMPip] [FuA] IL based SC has a broad and consistent potential range of up to 4 V, which is wider than the [EPy][FuA] IL (Figures S33a‐d).

During the cathodic sweep, the voltage of the working electrode in a supercapacitor gradually lowers to the negative range, resulting in an augmentation of ion deposition on the electrode surface (reduction phase). On the other hand, the potential of the electrode moves toward the positive range at the anodic sweep, causing the release of ions and oxidation of the species on the electrode surface. The increased potential range for SC with [EMPip] [FuA] IL is due to the presence of the 1‐ethyl‐1‐methylpiperidinium [EMPip] cation. This cation has a nonaromatic, fully saturated six‐membered ring structure and lacks π‐electron conjugation and aromaticity.^[^ ^62^ ^]^

Despite the relatively lower potential stability window of [EPy][FuA] IL, it offers a higher specific capacitance and reversibility at elevated temperatures due to its higher ionic conductivity and lower resistivity, as confirmed by the EIS data (Figures S33e and f).^[^ ^39^, ^41^ ^]^ The high ionic conductivity of the aromatic [EPy] cation allows fast ion movement, while the ethyl group offers further inductive stability, complementing the resonance effect and leading to a larger specific capacitance. In addition, the aromatic heterocyclic ring with a conjugated π‐system benefits from effective charge delocalization throughout the aromatic ring, and thus reducing the energy needed for charge storage.^[^ ^63^, ^64^ ^]^ Moreover, when voltage is applied, more charges accumulate on the electrodes, creating an electric field that attracts ions from the electrolyte to the electrode surface, lowering the internal resistance to the flow of electric current.^[^ ^39^, ^41^ ^]^ As more ions accumulate, the effective surface area available for charge storage increases, resulting in a decrease in the impedance.^[^ ^39^, ^41^ ^]^ Both the ILs exhibit straight lines in the lower frequency domain of the Nyquist plots, and hence capacitive behavior, while their redox behavior leads to uneven semicircles.^[^ ^41^, ^42^ ^]^ Moreover, the [EPy][FuA] IL with faster ion mobility minimizes the internal resistance ensuring the ions to readily access the electrode surface, and the lower internal resistance translates to improved efficiency and performance of the corresponding SC.^[^ ^41^, ^65^ ^]^

**Figure 9:**
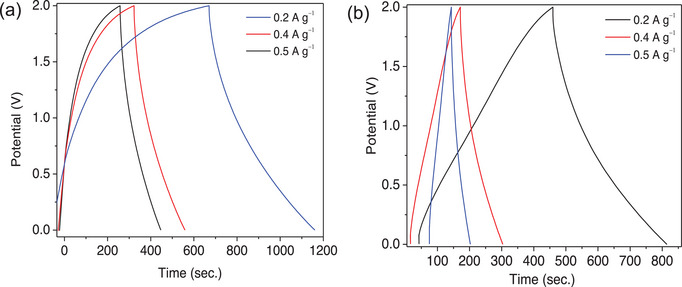
Galvanostatic charge‐discharge plots at 60 °C for the SCs with a) [EPy][FuA]; and b) [EMPip][FuA] electrolytes.

The SC using [EPy][FuA] IL as an electrolyte provided a specific capacitance of ∼241 F g^−1^ at 1 mV s^−1^ and 60 °C, which is significantly higher than that of SC with [EMPip][FuA] IL that displayed ∼139 F g^−1^ under the same conditions (Figure 8). The specific capacitance gradually decreases with increasing the scan rates and agrees well with the traditional double‐layer behavior.^[^ ^41^, ^65^ ^]^ This is due to the fact that the electrolyte ions have more time to rearrange and aggregate at the electrode‐electrolyte interface at slower scan rates, and leading to a thicker double layer.^[^ ^65^, ^66^ ^]^ Moreover, the charge‐discharge analysis revealed that the SC with [EPy][FuA] offered the highest specific capacitance of 99 F g^˗1^ and lowest specific capacitance of 89 F g^˗1^ at the current density of 0.2 A g^˗1^ and 0.5 A g^˗1^, respectively (Figure 9a and b). The capacitance of SC with [EPy][FuA] decreases with an increase in the current density and correlates well with the performance obtained through CV data (Figure 9a and Table S3). On the other hand, the SC with [EMPip][FuA] exhibit highest specific capacitance of 70 F g^˗1^ and lowest specific capacitance of 29 F g^˗1^ at the current densities of 0.2 A g^˗1^, and 0.5 A g^˗1^, respectively (Figure 9b and Table S3).

The charge‐discharge stability at each SC was validated by their capacitive performance and the double‐layer stability of charge‐discharge plots depicted by their triangular shape at all current densities (Figures 9a and b). During the charging process, ions diffuse through the electrolyte to reach the electrode‐electrolyte contact, whereas the process is reversed during the discharging.^[^ ^41^ ^]^ The faster ion diffusion allows quicker charge and discharge cycles and results in a higher power density. The impedance analysis of SC with [EPy][FuA] displayed sheets resistance of 7 Ω at 60 °C, which increased to 77 Ω combined with the resistance of the electrode and the electrolyte (70 Ω). However, the EIS analysis of the SC with [EMPip][FuA] displayed higher sheets resistance of 16 Ω at 60 °C and further increased to 117 Ω combined with the resistance of the electrode and the electrolyte (101 Ω) (Table S4).

**Figure 10:**
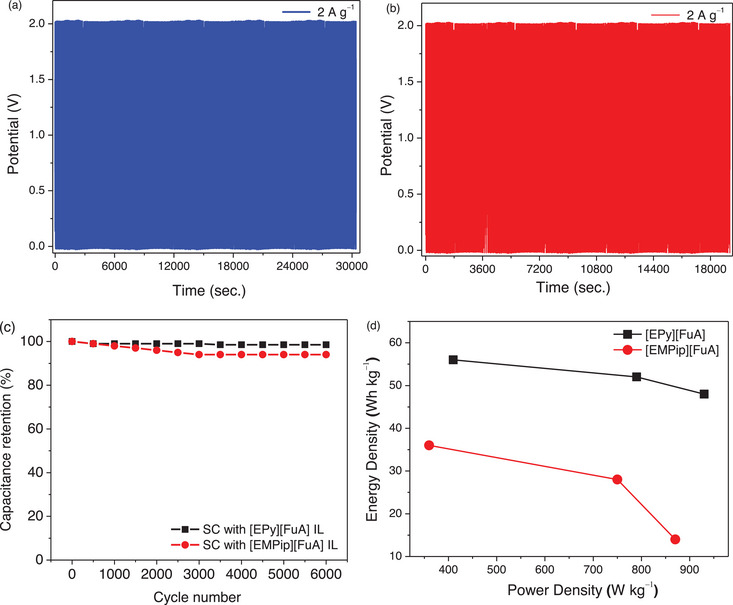
Long term stability of the SCs with a) [EPy][FuA] and b) [EMPip][FuA] electrolytes at 60 °C; c) Specific capacitance retention, d) Ragone plots depicting energy and power density of the [EPy][FuA] and [EMPip][FuA] IL‐based SCs at 60 °C.

The SCs offered excellent stability over many cycles enabled by electrochemical stability of the electrolyte and the electrode materials, as confirmed by the cyclic stability data (Figure 10a and 10b).^[^ ^65^, ^66^ ^]^ At 60 °C, the [EPy][FuA]‐IL‐based SC exhibit high cyclic stability of 6000 cycles at a current density of 2 A g^−1^ and more than 96% coulombic efficiency compared to the [EMPip][FuA]‐IL‐based SC, that has provided 93% coulombic efficiency under the same conditions (Figure S35). In addition, the SCs retained 98% and 94% of their initial capacitances after 6 000 cycles, for [EPy][FuA] and [EMPip][FuA] IL‐based SCs, respectively (Figure 10c), which is comparable with the previous studies based on aqueous electrolytes.^[^ ^66^, ^67^ ^]^ In terms of power and energy densities, the [EPy][FuA]‐IL‐based SC offered power density of 410 W kg^−1^ and energy density of 56 Wh kg^−1^ at 0.2 Ag^−1^, which are significantly higher than its structural analogous [EMPip][FuA] IL‐based electrolyte that provided only 360 W kg^−1^ and 36 Wh kg^−1^ (Figure 10d, Table S5). Altogether, the synthesized biomass‐derived fluorine‐free ILs offered a range of beneficial physical and electrochemical properties, however, their energy density performance in MWCNT‐based symmetric SCs is comparable/slightly lower than the SCs based on commercially available fluorinated ILs (Table S6). Since electrochemical performance of a supercapacitor is determined by the combination of electrolyte and electrode, therefore, the performance of these IL‐based electrolytes can be further improved and optimized by using other electrode materials.

## 4 Conclusions

A comprehensive structure‐property relationship for ten fluorine‐free biomass‐derived ILs with various structural designs of cations and anions is established. All the investigated ILs displayed low glass transition temperatures, high thermal stabilities, and desirable transport and electrochemical properties. Even though all the ILs remained in liquid phase over a wide range of temperature, their ion transport characteristics are strongly influenced by the cation's chemistry, size, and symmetry. The ionic conductivity is decreased by the length of alkyl chains on the cations as well as the asymmetrical ring sizes due to their reduced rotational flexibility and enhanced ionic interactions. The ion mobilities decreased with increasing cation sizes and cation‐anion interactions, and the latter decreased by increasing temperature to achieve higher ionic mobilities, as confirmed by FTIR and NMR spectroscopy. The [EPy][FuA] IL electrolyte offered higher specific capacitance and better long‐term stability in a symmetric supercapacitor based on MWCNT than its structural analogous [EMPip][FuA] IL. This study demonstrates a step towards the development of greener and sustainable fluorine‐free ionic liquid electrolytes for supercapacitors operating at elevated temperatures.

## Conflict of Interest

The authors declare no conflict of interest.

## Supporting information



Supporting Information

## Data Availability

The data that support the findings of this study are available in the supplementary material of this article.
