# Supplementary material for: Fluorine‐Free Biomass‐Derived Ionic Liquid Electrolytes: Ion Dynamics and Electrochemical Properties
Source: Chemistry. 2025 Aug 20;31(53):e01641. doi: 10.1002/chem.202501641 (PMC12451414; doi:10.1002/chem.202501641)
Supplement: Supplementary file 1 — Supporting Information [file CHEM-31-e01641-s001.docx]

**Supporting Information**

**Fluorine-Free** **Biomass-Derived Ionic Liquid Electrolytes: Ion Dynamics and Electrochemical Properties**

**Sayantika Bhakta, Gaurav Tatrari, Maiia Rudakova, Andrei Filippov, and Faiz Ullah Shah^*^**

Chemistry of Interfaces, Luleå University of Technology, SE-971 87, Luleå, Sweden

^*^Corresponding author:

[faiz.ullah@ltu.se](mailto:faiz.ullah@ltu.se)

**Table of Content**

Synthesis and characterization of ionic liquids ……………………………………………...2-6

Figures S1–S10. ESI-MS spectra…………………………………………………………...7-11

Figures S11–S30. ^1^H and ^13^C NMR spectra……………………….……………………....12-21

Figure S-31. Nyquist plots of (a) neat ILs and (b) [BMPyrr][FuA] …………………………...22

Figure S32. CV plots at different scan rates at 30 °C for SCs………………………………….22

Figure S33. CV plots and Nyquist plots of the supercapacitors………………………………..23

Figure S34. Specific capacitance of the supercapacitors………………………………………24

Figure S35. Coulombic efficiency of the supercapacitors……………………………………..24

Table S1. VFT equation parameters of the ionic conductivity ……………………..………….25

Table S2. VFT equation parameters of the ion diffusion data……………………….………...25

Table S3. Specific capacitance of the supercapacitors………………………………………...26

Table S4. The EIS data-based resistance values of the supercapacitors …………………..…..26

Table S5. Energy and power density of the supercapacitors…………………………………..26

Table S6. Energy density evaluated for reported IL-based SC respectively …………………..26

References…………………………………………………………………………………….27

**Synthesis of ionic liquids**

As an example, the synthesis procedure for 1-butyl-1-methylpyrrolium furoate [BMPyrr][FuA] is described in detail here (Scheme S1), while all the other ionic liquids are synthesized using the same reaction procedure.

**Step 1:** In the first step, 1-butyl-1-methylpyrrolium bromide [BMPyrr]Br is synthesized by adding dropwise a solution of alkyl halide (1.2 equiv.) to 1-methylpyrrolidine (1 equiv.) under cooling condition. The reaction mixture was stirred at room temperature for 24 hours and after completion, the reaction mixture was washed with ethyl acetate to get the desired white solid product of [BMPyrr]Br. The product was dried in a vacuum oven at 80 ^o^C for over 3 days before using in the next step.

**Step 2:** In the second step, 1-butyl-1-methylpyrrolium hydroxide [BMPyrr]OH is synthesized by adding silver oxide (1.18 g, 5.1 mmol, 0.51 equiv.) to the aqueous solution of the [BMPyrr]Br (2.21 g, 10 mmol, 1 equiv.). The reaction mixture was stirred for 2 hours, and this reaction mixture was filtered and used in the final step of synthesis of ionic liquids without any further purifications.

**Step 3:** In the final step, the ionic liquid was prepared by mixing the reaction mixture with an aqueous solution of furoic acid (1.34g, 12 mmol, 1.2 equiv.) and stirred overnight at room temperature. After completion, water was rotary evaporated, and the product was washed with diethyl ether to remove the unreacted acid. The final product was extracted with dichloromethane and dried over sodium sulphate to remove traces of water. The product was filtered, and the solvent was evaporated using a rotary evaporator under reduced pressure to obtain the ionic liquid product. All the ionic liquid products were dried in a vacuum oven at 80 °C for about 7 days. All the products were separated into quantitative yields (91-95%) and characterized using NMR spectroscopy. The characterization detail for each ionic liquid is provided below.

**Scheme S1.** General synthetic outline of the synthesized ILs.

**[BMPyrr][FuA]:** Brown liquid, 95% yield.

MS (ESI) calculated for Chemical Formula: [C_9_H_20_N]^+^, calculated m/z: 142.1591, found m/z 142.1569; [C_5_H_3_O_3_]^-^, calculated m/z: 111.0087, found m/z 111.0146.

^1^H NMR (400 MHz, D_2_O) δ 7.50 (s, 1H), 6.91 (d, *J* = 3.1 Hz, 1H), 6.46 (d, *J* = 1.4 Hz, 1H), 3.34 (t, *J* = 18.0 Hz, 4H), 3.25 – 3.11 (m, 2H), 2.91 (s, 3H), 2.09 (s, 4H), 1.65 (dt, *J* = 15.7, 7.9 Hz, 2H), 1.36 – 1.16 (m, 2H), 0.84 (t, *J* = 7.4 Hz, 3H).

^13^C NMR (101 MHz, D_2_O) δ 166.58, 149.49, 145.22, 115.13, 111.80, 64.39, 48.27, 48.23, 25.25, 21.49, 19.41, 12.96.

**[BMPyrr][HFuA]:**

The procedure is similar to that used in the synthesis of [BMPyrr][FuA]. The desired ionic liquid was synthesized by the reaction of [BMPyrr]Br (2.21 g, 10 mmol), silver (I) oxide (1.18 g, 5.1 mmol), tetrahydrofuroic acid (1.392 g, 12 mmol). A viscous brown ionic liquid was obtained in 94 % yield.

MS (ESI) calculated for Chemical Formula: [C_7_H_16_N]^+^, calculated m/z: 142.1591, found m/z 142.1582; [C_5_H_7_O_3_]^-^, calculated m/z: 115.0400, found m/z 115.365.

^1^H NMR (400 MHz, D_2_O) δ 4.20 (d, *J* = 6.3 Hz, 1H), 3.91 – 3.81 (m, 1H), 3.76 (d, *J* = 6.4 Hz, 1H), 3.40 (s, 4H), 3.27 – 3.17 (m, 2H), 2.94 (s, 3H), 2.26 – 2.00 (m, 4H), 1.89 – 1.74 (m, 4H), 1.69 (dd, *J* = 15.6, 8.0 Hz, 2H), 1.30 (dd, *J* = 14.7, 7.4 Hz, 2H), 0.86 (t, *J* = 7.3 Hz, 3H).

^13^C NMR (101 MHz, D_2_O) δ 181.29, 78.46, 69.08, 64.42, 48.27, 30.63, 25.22, 21.49, 19.42, 12.96.

**[EMPyrr][FuA]:**

The procedure is similar to that used in the synthesis of [BMPyrr][FuA]. The desired ionic liquid was synthesized by the reaction of [EMPyrr]Br (1.94 g, 10 mmol), silver (I) oxide (1.18 g, 5.1 mmol), Furoic acid (1.34 g, 12 mmol). A viscous pale brown ionic liquid was obtained in 95 % yield.

MS (ESI) calculated for Chemical Formula: [C_7_H_16_N]^+^, calculated m/z: 114.1278, found m/z 114.1275; [C_5_H_3_O_3_]^-^, calculated m/z: 111.0087, found m/z 111.0143.

^1^H NMR (400 MHz, D_2_O) δ 7.49 (s, 1H), 6.93 (d, *J* = 3.3 Hz, 1H), 6.44 (dd, *J* = 3.2, 1.6 Hz, 1H), 3.29 (d, *J* = 4.0 Hz, 3H), 3.22 (dd, *J* = 14.5, 7.3 Hz, 2H), 2.83 (s, 3H), 1.98 (d, *J* = 31.5 Hz, 4H), 1.47 – 0.71 (m, 3H).

^13^C NMR (101 MHz, D_2_O) δ 165.68, 148.49, 145.70, 115.97, 111.96, 63.93, 63.90, 59.69, 47.63, 47.59, 21.47, 8.59.

**[EPy][FuA]:**

The procedure is similar to that used in the synthesis of [BMPyrr][FuA]. The desired ionic liquid was synthesized by the reaction of [EPy]Br (1.88 g, 10 mmol), silver (I) oxide (1.18 g, 5.1 mmol), Furoic acid (1.34 g, 12 mmol). A viscous brown ionic liquid was obtained in 94 % yield.

MS (ESI) calculated for Chemical Formula: [C_7_H_10_N]^+^, calculated m/z: 108.0808, found m/z 108.0812; [C_5_H_3_O_3_]^-^, calculated m/z: 111.0087, found m/z 111.0162.

^1^H NMR (400 MHz, D_2_O) δ 8.73 (d, *J* = 5.6 Hz, 2H), 8.40 (t, *J* = 7.6 Hz, 1H), 7.93 (s, 2H), 7.47 (s, 1H), 6.90 (d, *J* = 3.2 Hz, 1H), 6.42 (s, 1H), 4.52 (dd, *J* = 14.5, 7.2 Hz, 2H), 1.48 (dd, *J* = 36.0, 28.9 Hz, 3H).

^13^C NMR (101 MHz, D_2_O) δ 165.87, 148.55, 145.62, 144.14, 144.05, 143.97, 128.40, 115.84, 111.88, 57.49, 15.74.

**[BPy][FuA]:**

The procedure is similar to that used in the synthesis of [BMPyrr][FuA]. The desired ionic liquid was synthesized by the reaction of [BPy]Br (2.16 g, 10 mmol), silver (I) oxide (1.18 g, 5.1 mmol), Furoic acid (1.34 g, 12 mmol). A viscous brown ionic liquid was obtained in 95 % yield.

MS (ESI) calculated for Chemical Formula: [C_9_H_14_N]^+^, calculated m/z: 136.1121, found m/z 136.1145; [C_5_H_3_O_3_]^-^, calculated m/z: 111.0087, found m/z 111.0167.

^1^H NMR (400 MHz, D_2_O) δ 8.72 (d, *J* = 5.5 Hz, 2H), 8.42 (t, *J* = 7.6 Hz, 1H), 7.94 (s, 2H), 7.50 (s, 1H), 6.93 (d, *J* = 3.3 Hz, 1H), 6.46 (s, 1H), 4.49 (t, *J* = 7.3 Hz, 2H), 2.07 – 1.67 (m, 2H), 1.43 – 1.07 (m, 2H), 0.95 – 0.59 (m, 3H).

^13^C NMR (101 MHz, D_2_O) δ 165.92, 148.65, 145.59, 144.31, 144.23, 128.35, 115.79, 111.90, 61.90, 32.69, 18.86, 12.78.

**[EMPip][FuA]:**

The procedure is similar to that used in the synthesis of [BMPyrr][FuA]. The desired ionic liquid was synthesized by the reaction of [EMPip]Br (2.08 g, 10 mmol), silver (I) oxide (1.18 g, 5.1 mmol), Furoic acid (1.34 g, 12 mmol). A viscous slightly brownish ionic liquid was obtained in 91 % yield.

MS (ESI) calculated for Chemical Formula: [C_8_H_18_N]^+^, calculated m/z: 128.1434, found m/z 128.1431; [C_5_H_3_O_3_]^-^, calculated m/z: 111.0087, found m/z 111.0162.

^1^H NMR (400 MHz, D_2_O) δ 7.50 (s, 1H), 6.91 (d, *J* = 3.2 Hz, 1H), 6.46 (s, 1H), 3.27 (dd, *J* = 14.4, 7.2 Hz, 2H), 3.21 – 3.02 (m, 4H), 2.86 (s, 3H), 1.74 (s, 4H), 1.63 – 1.41 (m, 2H), 1.20 (t, *J* = 5.5 Hz, 3H).

^13^C NMR (101 MHz, D_2_O) δ 166.47, 149.33, 145.30, 115.27, 111.83, 60.77, 59.07, 47.09, 20.77, 19.65, 6.82.

**[BMPip][FuA]:**

The procedure is similar to that used in the synthesis of [BMPyrr][FuA]. The desired ionic liquid was synthesized by the reaction of [BMPip]Br (2.36 g, 10 mmol), silver (I) oxide (1.18 g, 5.1 mmol), Furoic acid (1.34 g, 12 mmol). A viscous slightly brownish ionic liquid was obtained in 90 % yield.

MS (ESI) calculated for Chemical Formula: [C_10_H_22_N]^+^, calculated m/z: 156.1747, found m/z 156.1731; [C_5_H_3_O_3_]^-^, calculated m/z: 111.0087, found m/z 111.0162.

^1^H NMR (400 MHz, D_2_O) δ 7.49 (s, 1H), 6.90 (d, *J* = 3.3 Hz, 1H), 6.45 (dd, *J* = 2.9, 1.5 Hz, 1H), 3.16 (d, *J* = 16.6 Hz, 6H), 2.87 (s, 3H), 1.73 (s, 4H), 1.65 – 1.43 (m, 4H), 1.37 – 1.14 (m, 2H), 0.83 (t, *J* = 7.3 Hz, 3H).

^13^C NMR (101 MHz, D_2_O) δ 166.46, 149.34, 145.29, 115.25, 111.82, 63.44, 61.24, 47.80, 23.26, 20.76, 19.68, 19.37, 12.98.

**[EMMIm][FuA]:**

The procedure is similar to that used in the synthesis of [BMPyrr][FuA]. The desired ionic liquid was synthesized by the reaction of [EMMIm]Br (2.08 g, 10 mmol), silver (I) oxide (1.18 g, 5.1 mmol), Furoic acid (1.34 g, 12 mmol). A viscous pale brown ionic liquid was obtained in 92 % yield.

MS (ESI) calculated for Chemical Formula: [C_8_H_18_N]^+^, calculated m/z: 128.1434, found m/z 128.1431; [C_5_H_3_O_3_]^-^, calculated m/z: 111.0087, found m/z 111.0172.

^1^H NMR (400 MHz, D_2_O) δ 7.50 (s, 1H), 7.19 (d, *J* = 14.8 Hz, 1H), 7.15 (d, *J* = 9.6 Hz, 1H), 6.93 (d, *J* = 3.1 Hz, 1H), 6.45 (d, *J* = 1.5 Hz, 1H), 3.99 (q, *J* = 7.3 Hz, 2H), 3.62 (s, 3H), 2.44 (s, 3H), 1.29 (t, *J* = 7.3 Hz, 3H).

^13^C NMR (101 MHz, D_2_O) δ 165.88, 148.62, 145.60, 122.25, 120.15, 115.77, 111.88, 43.47, 34.57, 14.22, 8.72.

**[BMMIm][FuA]:** The procedure is similar to that used in the synthesis of [BMPyrr][FuA]. The desired ionic liquid was synthesized by the reaction of [BMMIm]Br (2.33 g, 10 mmol), silver (I) oxide (1.18 g, 5.1 mmol), Furoic acid (1.34 g, 12 mmol). A viscous redish brown ionic liquid was obtained in 94 % yield.

MS (ESI) calculated for Chemical Formula: [C_7_H_13_N_2_]^+^, calculated m/z: 125.1074, found m/z 125.1090; [C_5_H_3_O_3_]^-^, calculated m/z: 111.0087, found m/z 111.0144.

^1^H NMR (400 MHz, D_2_O) δ 7.46 (s, 1H), 7.17 (s, 1H), 7.14 (s, 1H), 6.87 (d, *J* = 3.3 Hz, 1H), 6.42 (s, 1H), 4.70 (s, 3H), 3.93 (t, *J* = 7.2 Hz, 2H), 3.60 (s, 3H), 2.41 (s, 3H), 1.71 – 1.50 (m, 2H), 1.17 (dq, *J* = 14.5, 7.2 Hz, 2H), 0.78 (t, *J* = 7.3 Hz, 3H).

^13^C NMR (101 MHz, D_2_O) δ 166.19, 149.12, 145.35, 144.21, 122.20, 120.84, 115.35, 111.81, 48.07, 34.63, 31.14, 19.04, 12.90, 8.87.

**[BOMMIm][FuA]:**

The procedure is similar to that used in the synthesis of [BMPyrr][FuA]. The desired ionic liquid was synthesized by the reaction of [BOMMIm]Br (2.35 g, 10 mmol), silver (I) oxide (1.18 g, 5.1 mmol), Furoic acid (1.34 g, 12 mmol). A viscous redish brown ionic liquid was obtained in 93 % yield.

MS (ESI) calculated for Chemical Formula: [C_8_H_15_N_2_O]^+^, calculated m/z: 155.1179, found m/z 155.1147; [C_5_H_3_O_3_]^-^, calculated m/z: 111.0087, found m/z 111.0167.

^1^H NMR (400 MHz, D_2_O) δ 7.48 (s, 1H), 7.22 (d, *J* = 9.5 Hz, 1H), 7.19 (s, 1H), 6.89 (d, *J* = 3.2 Hz, 1H), 6.43 (s, 1H), 4.25 – 4.07 (m, 2H), 3.71 – 3.65 (m, 2H), 3.63 (s, 3H), 3.23 (s, 3H), 2.45 (s, 3H).

^13^C NMR (101 MHz, D_2_O) δ 166.36, 149.16, 145.36, 145.01, 122.47, 121.05, 115.35, 111.81, 70.11, 58.52, 47.69, 34.79, 9.08.


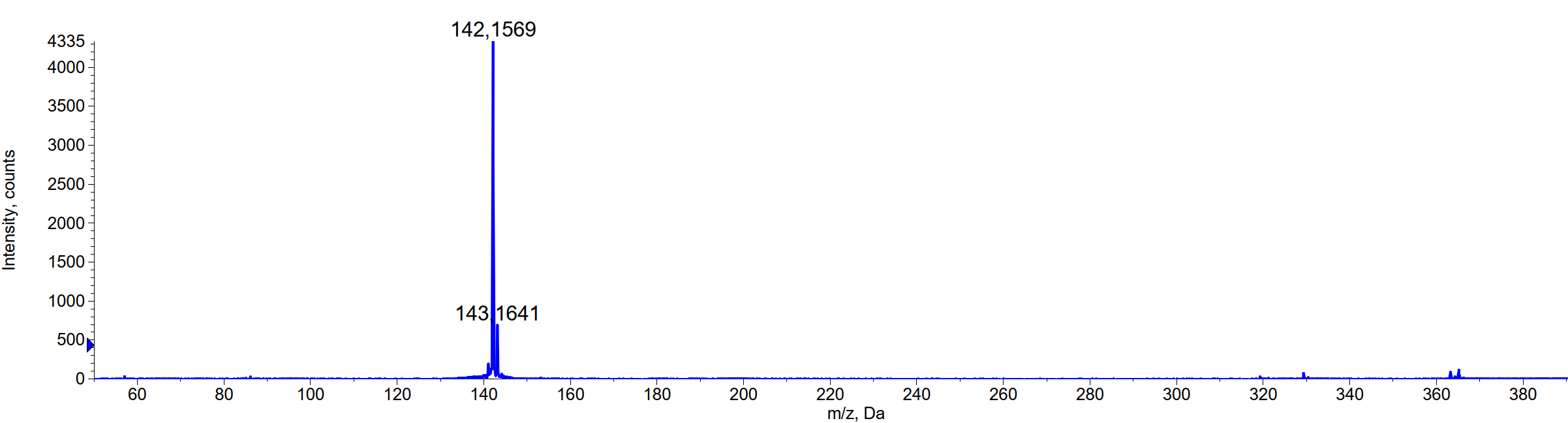


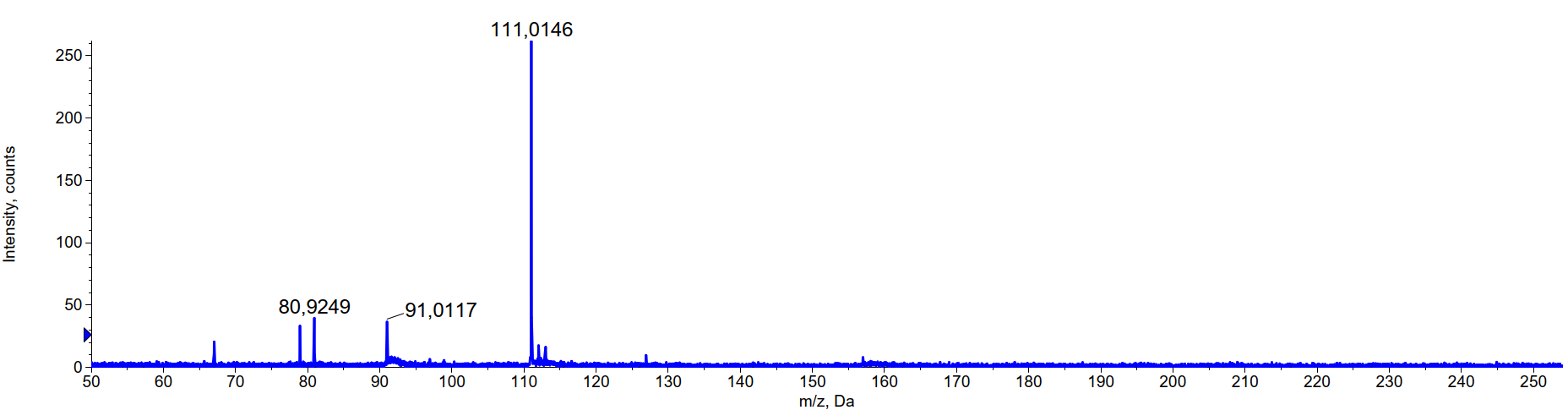


**Figure S1.** MS (ESI) of [BMPyrr][FuA].


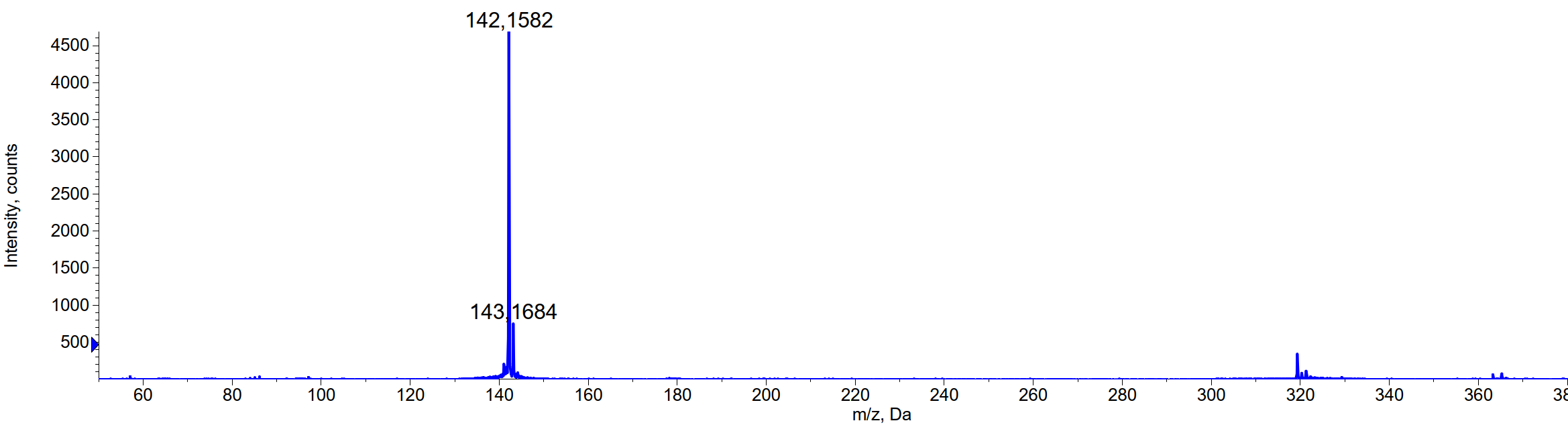


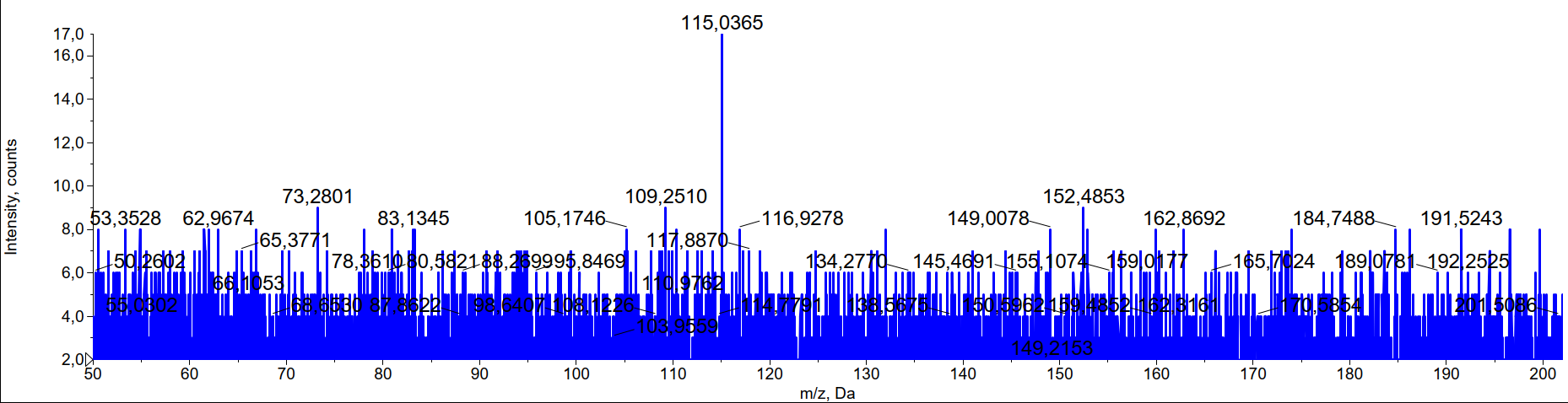


**Figure S2.** MS (ESI) of [BMPyrr][HFuA].


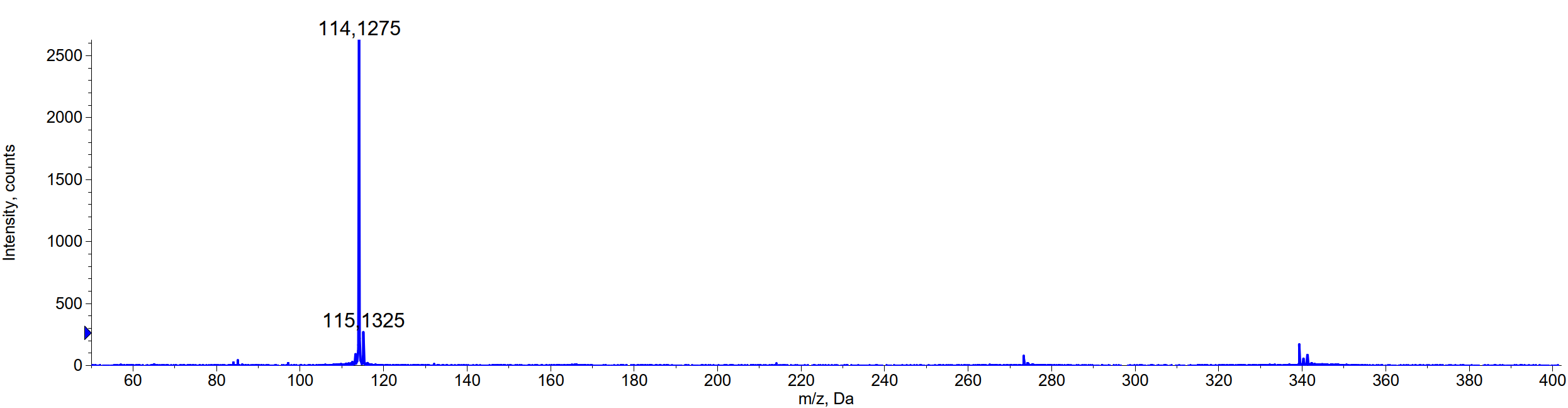


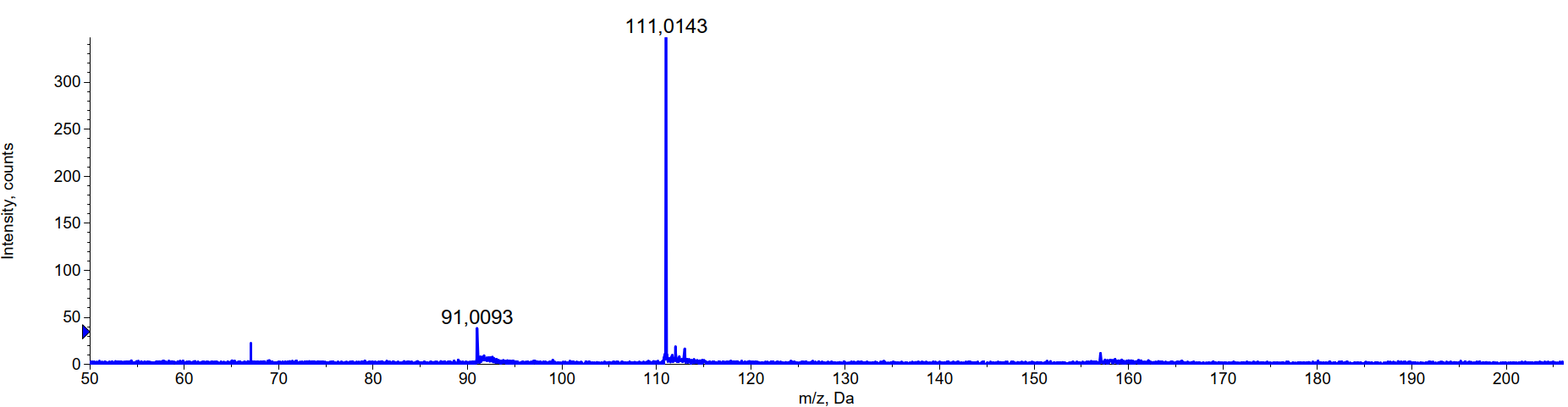


**Figure S3.** MS (ESI) of [EMPyrr][FuA].


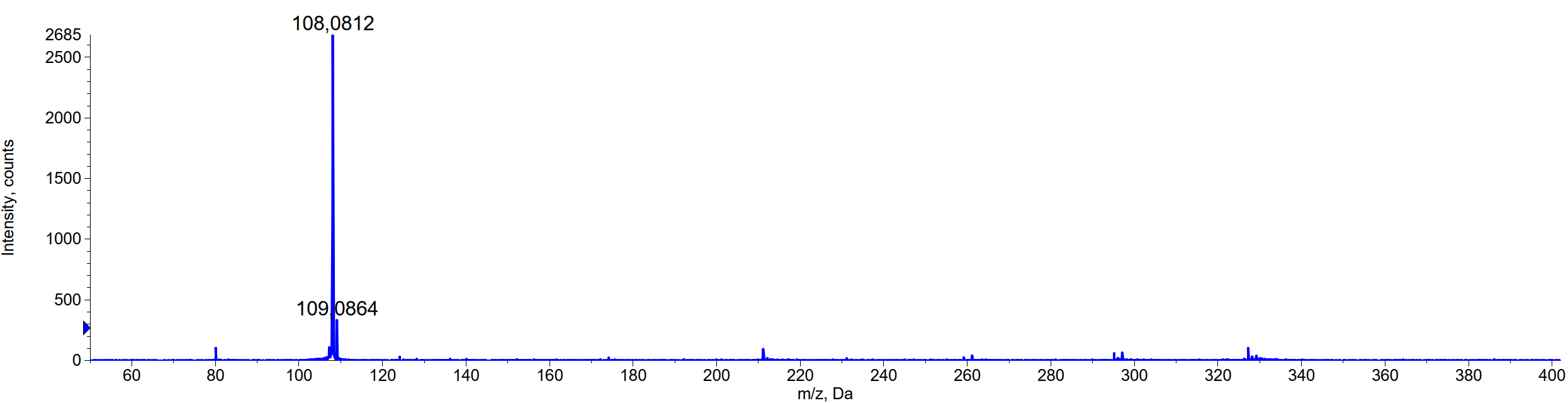


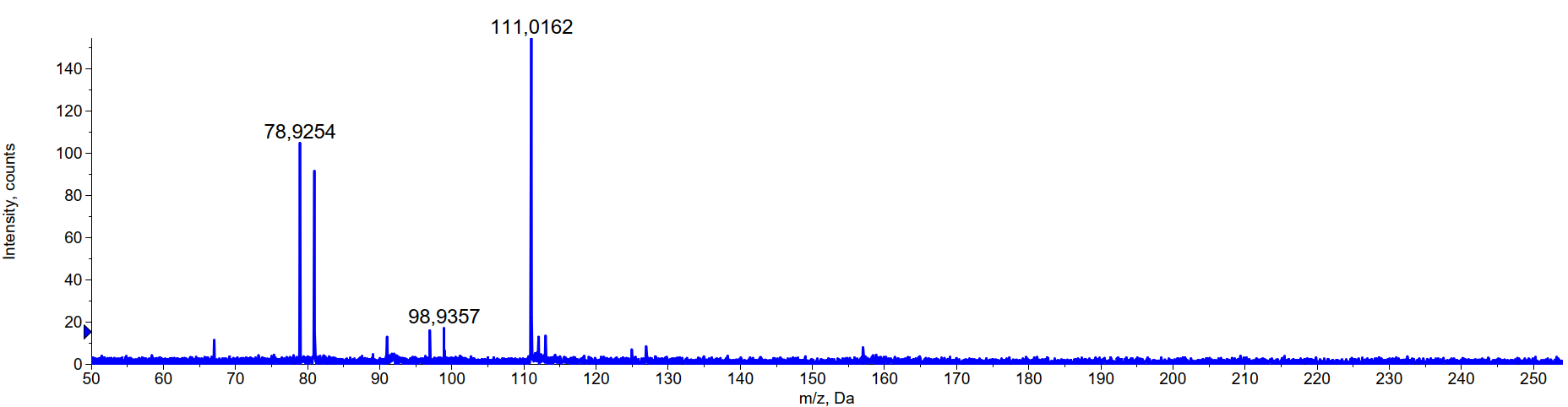


**Figure S4.** MS (ESI) of [EPy][FuA].


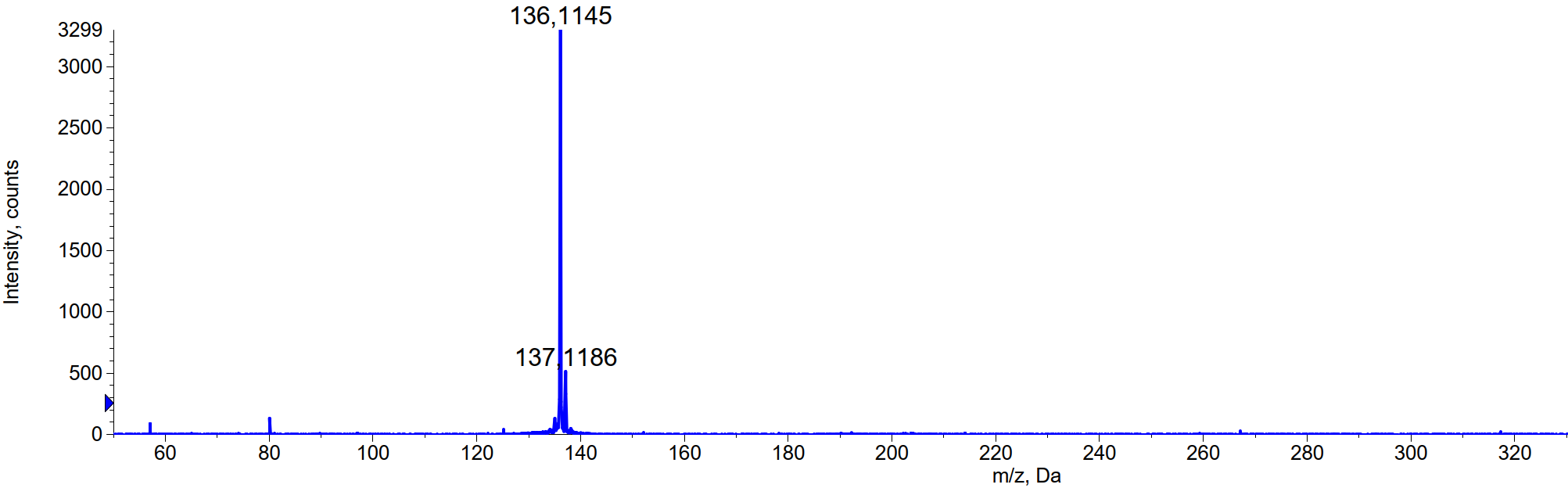


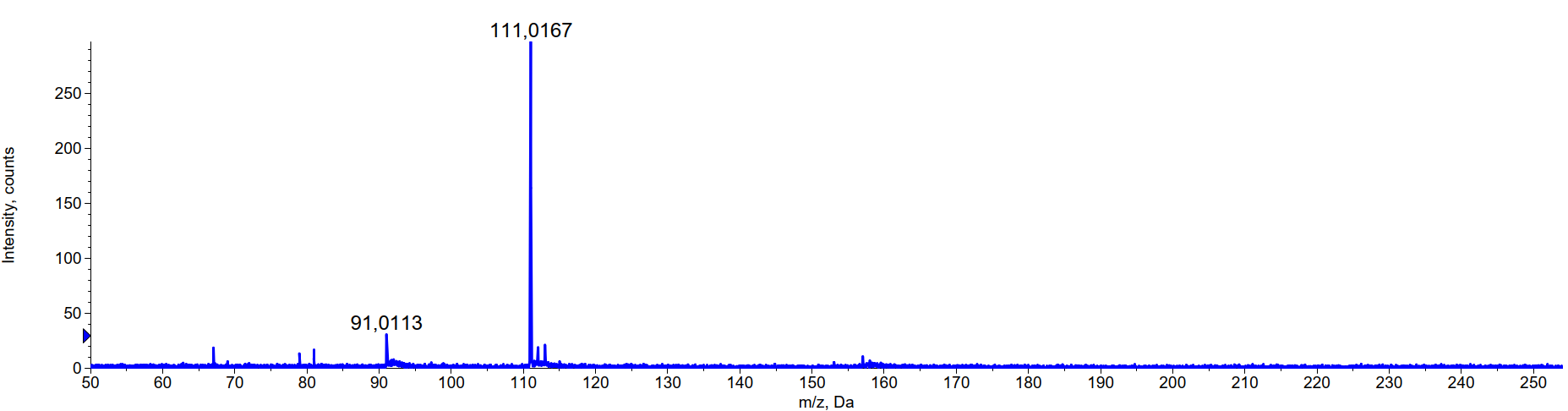


**Figure S5.** MS (ESI) of [BPy][FuA].


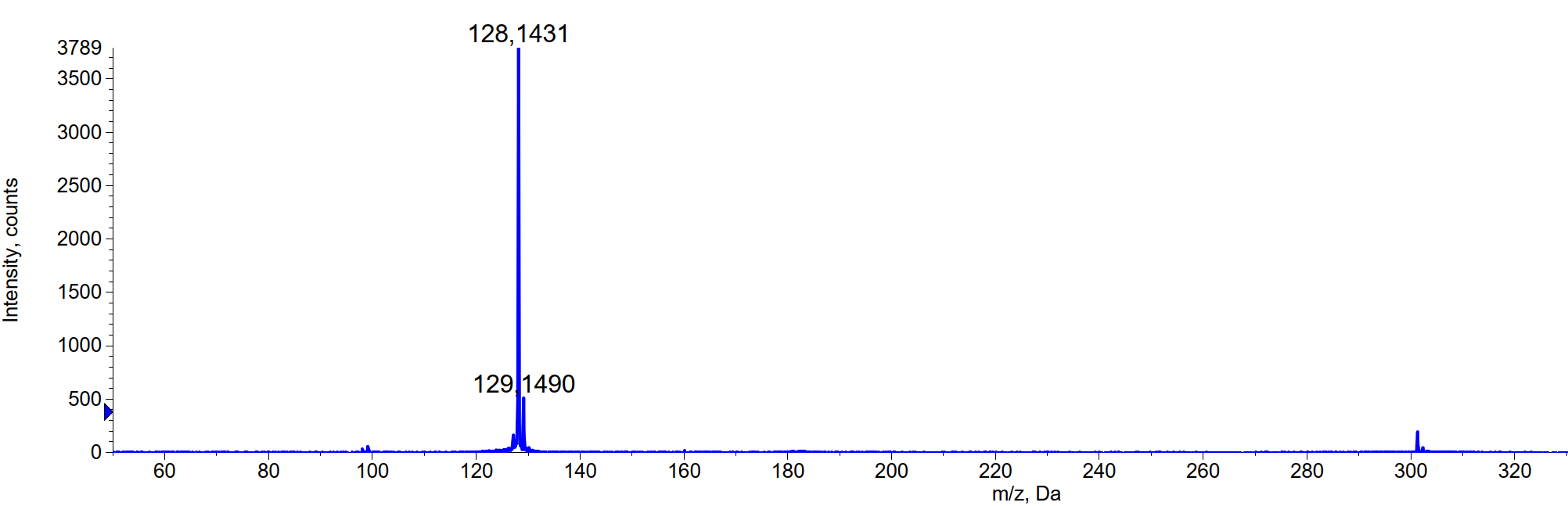


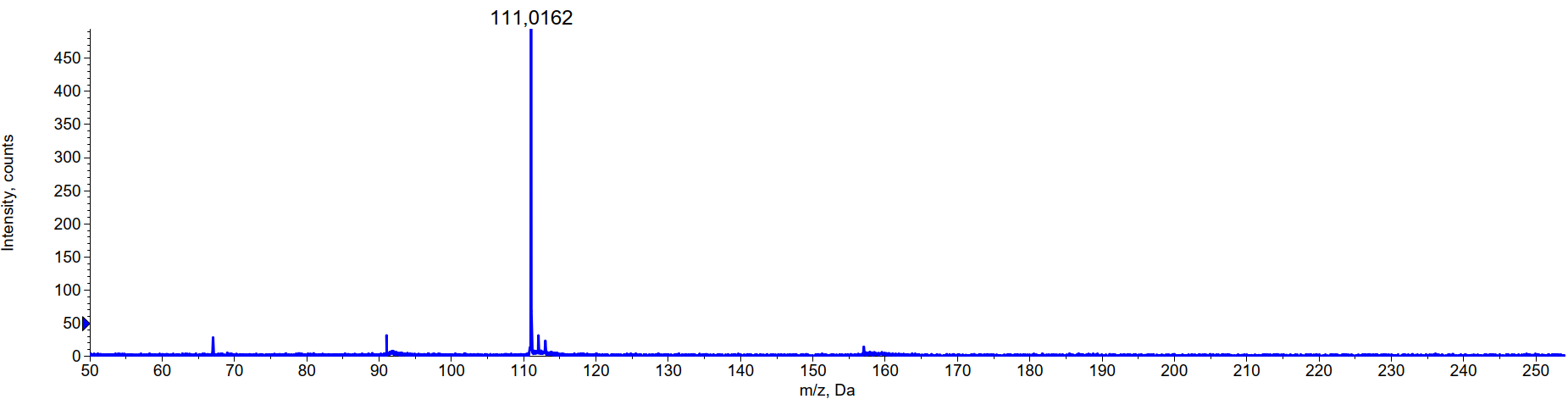


**Figure S6.** MS (ESI) of [EMPip][FuA].


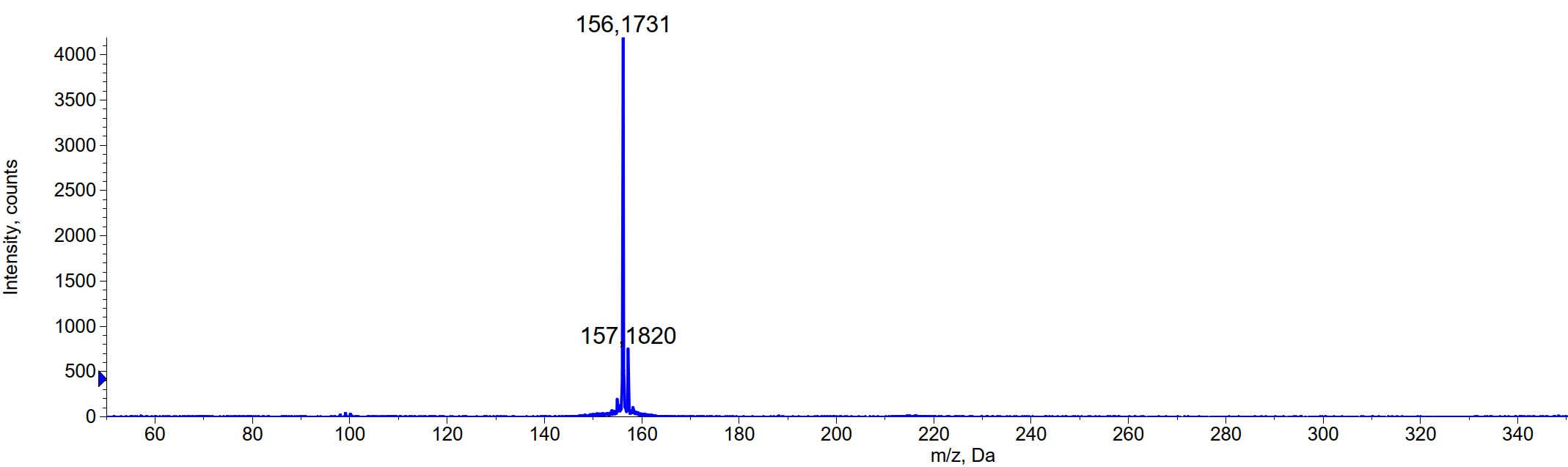


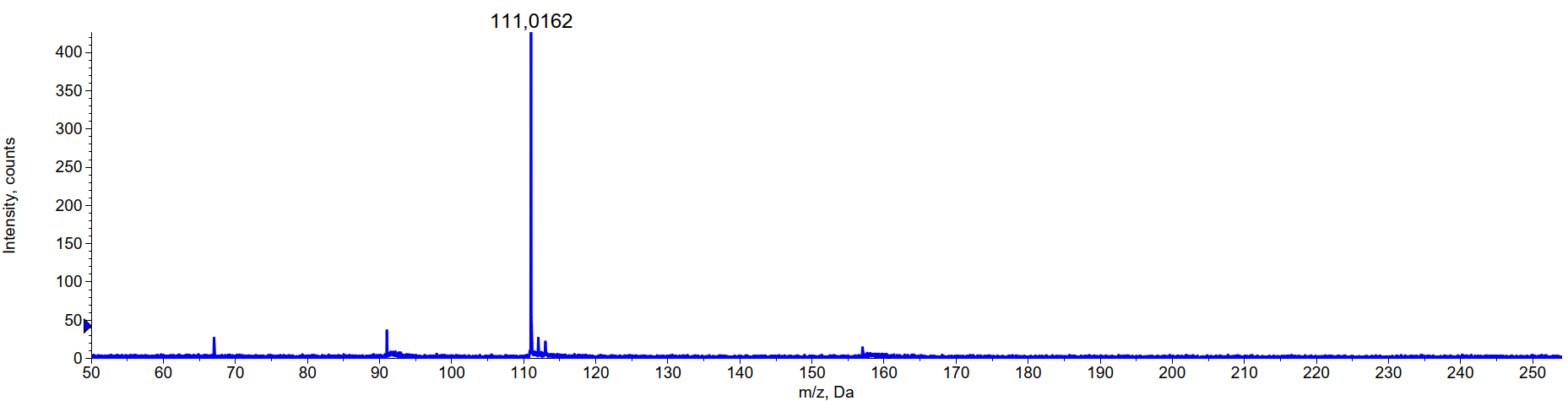


**Figure S7.** MS (ESI) of [BMPip][FuA].


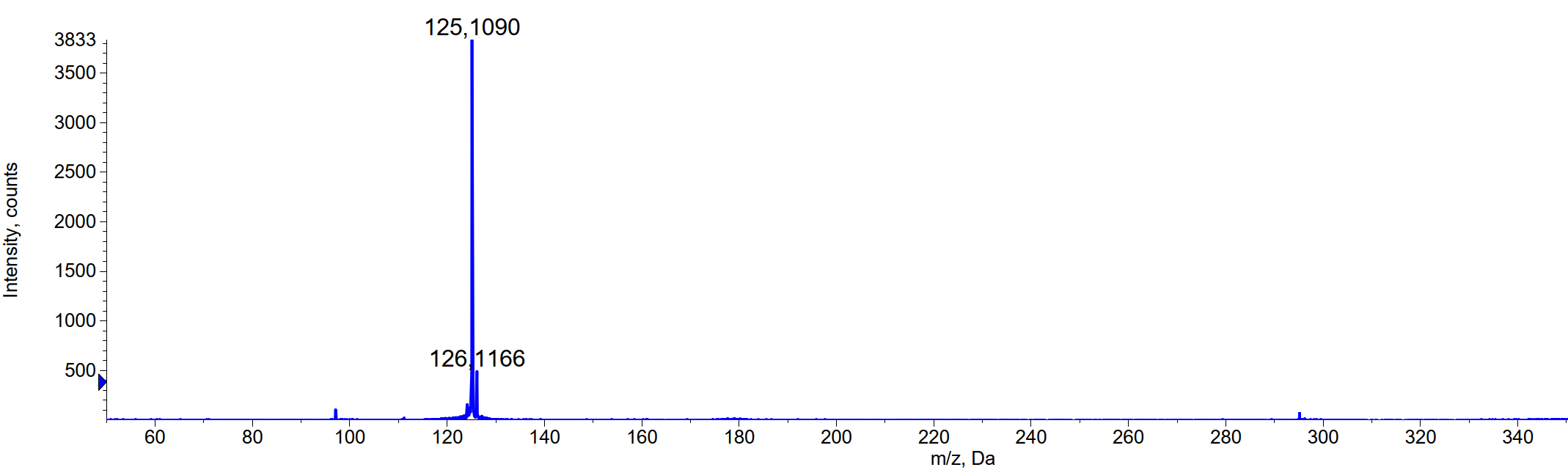


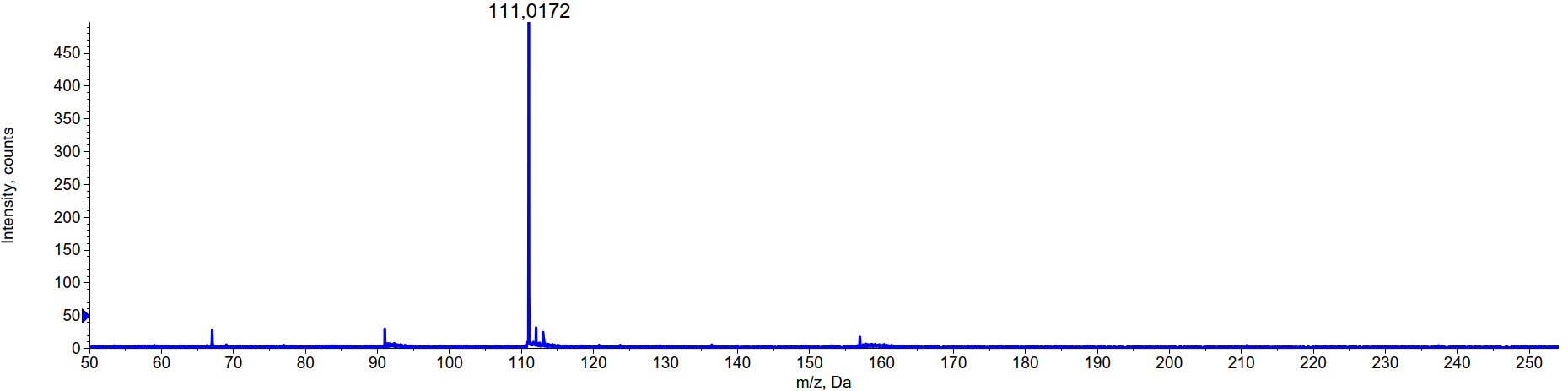


**Figure S8.** MS (ESI) of [EMMIm][FuA].


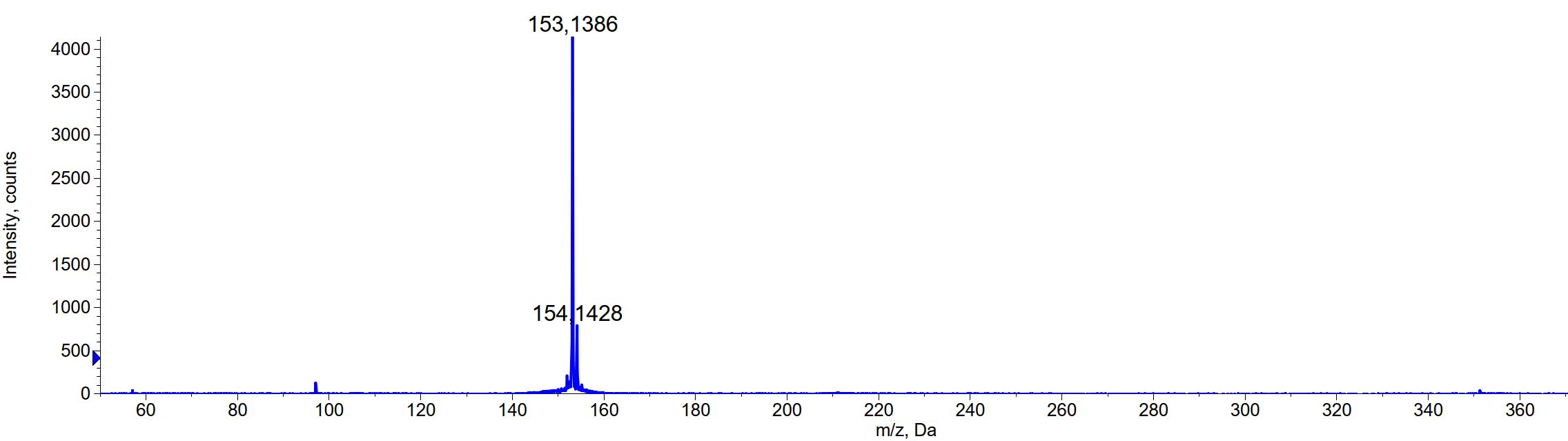


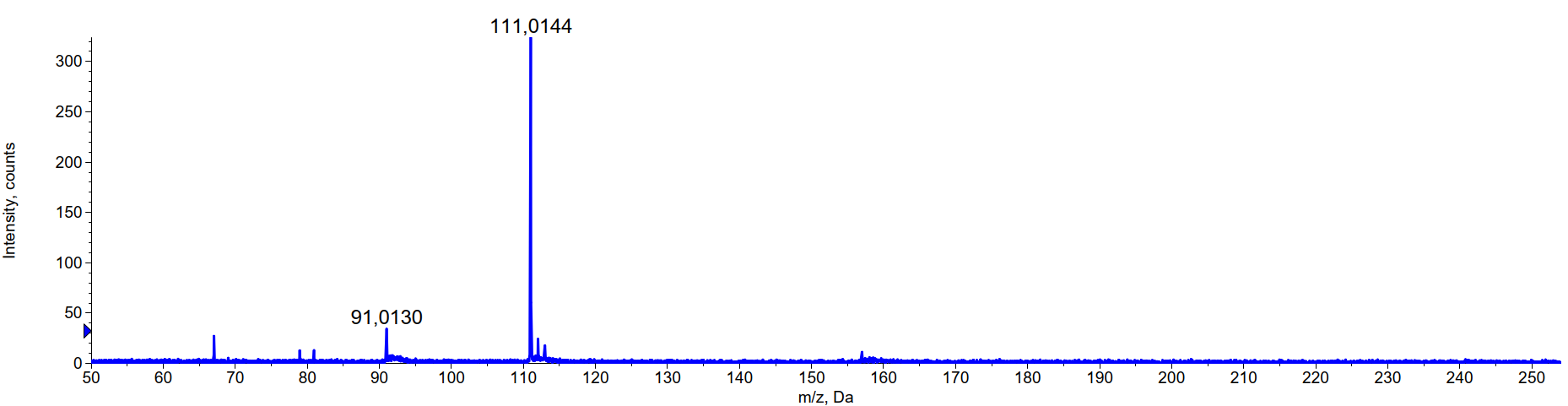


**Figure S9.** MS (ESI) of [BMMIm][FuA].


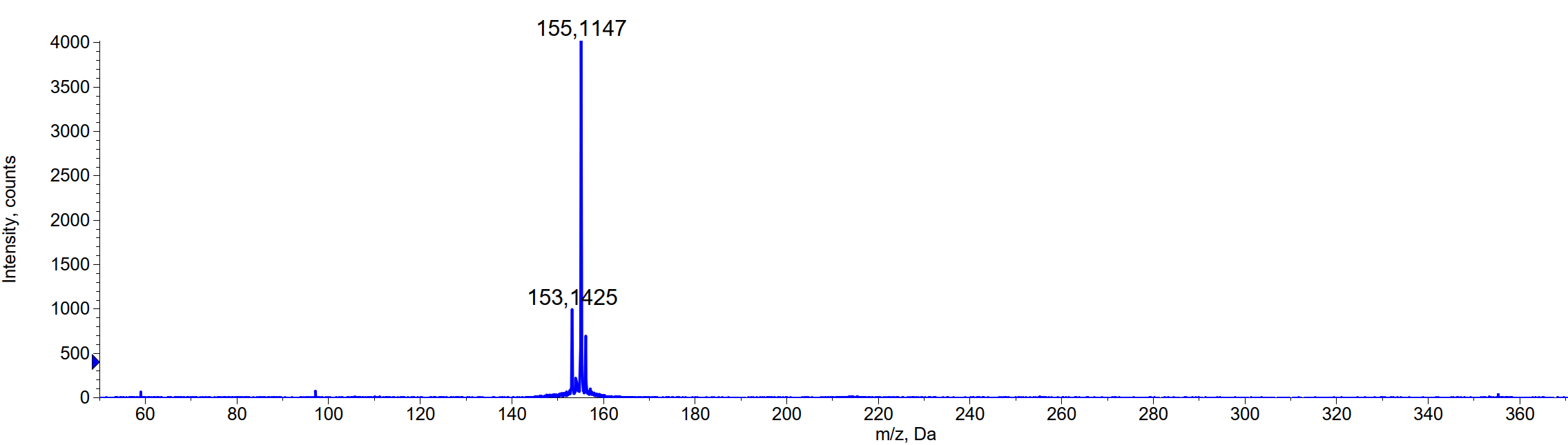


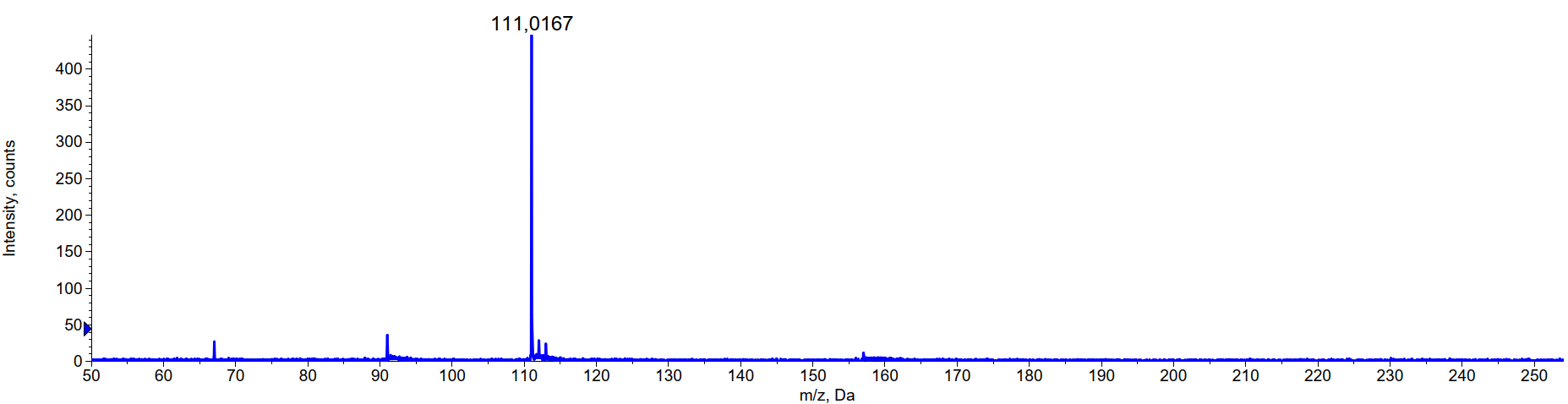


**Figure S10.** MS (ESI) of [BOMMIm][FuA].

**
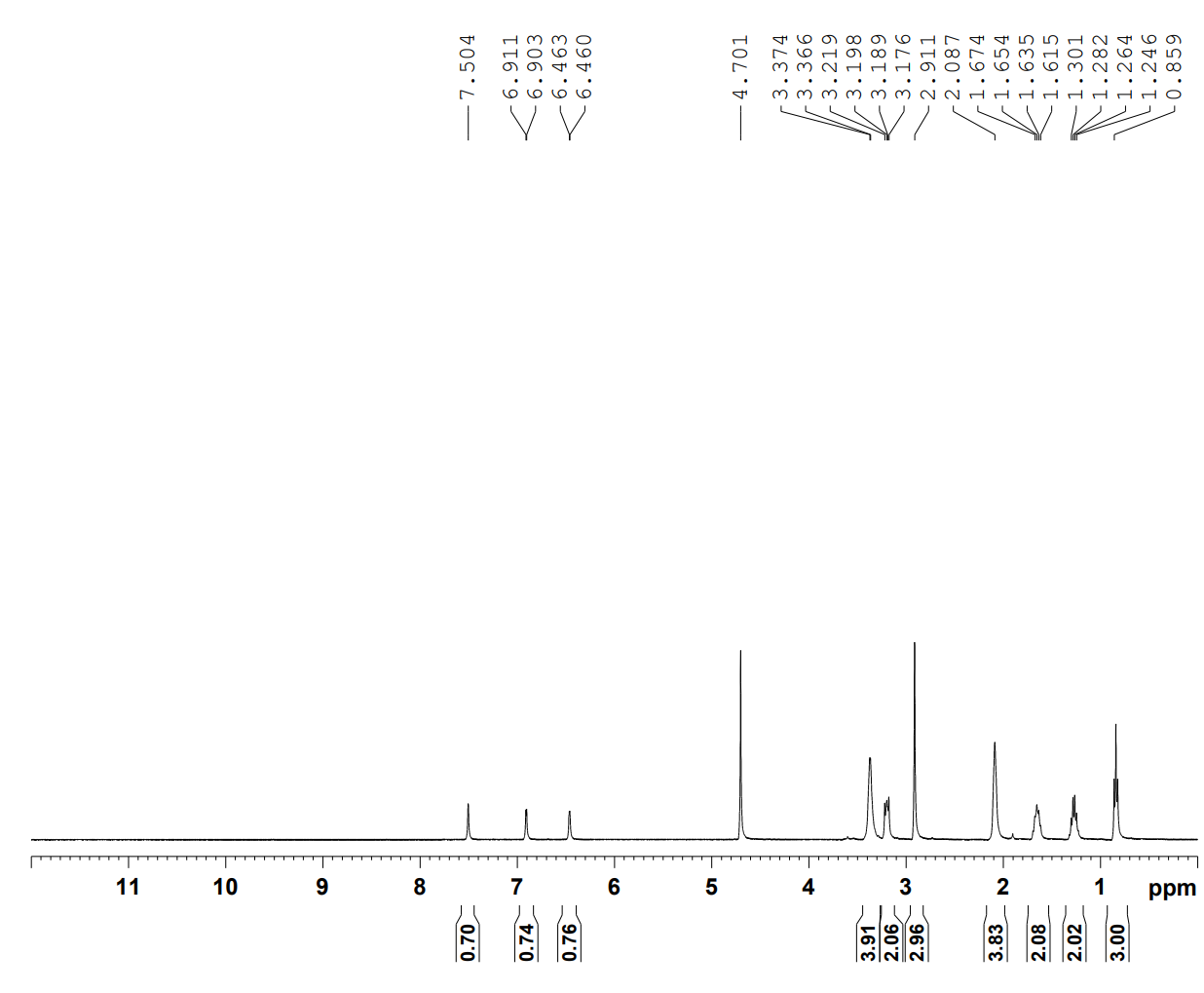
**

**Figure S11.** ^1^H spectrum of [BMPyrr][FuA] in D_2_O.

**
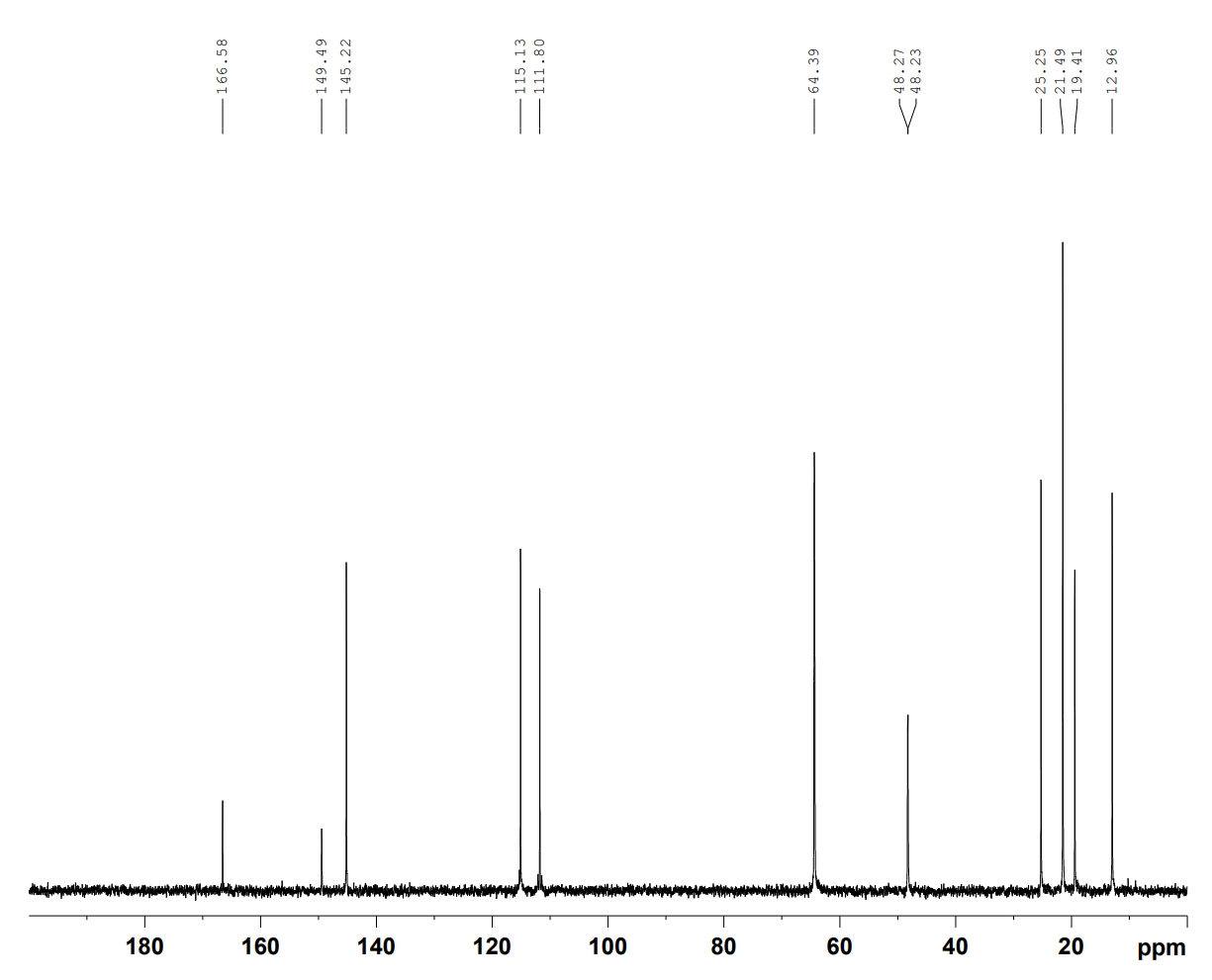
**

**Figure S12.** ^13^C spectrum of [BMPyrr][FuA] in D_2_O.

**
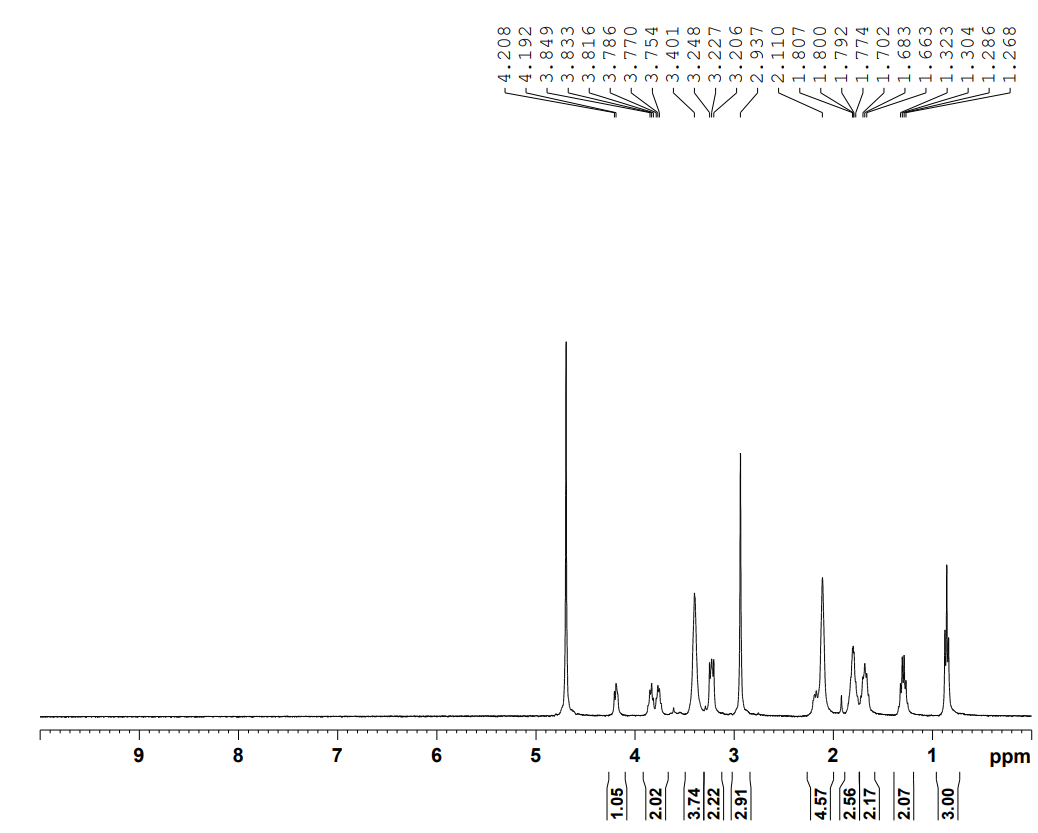
**

**Figure S13.** ^1^H spectrum of [BMPyrr][HFuA] in D_2_O.

**
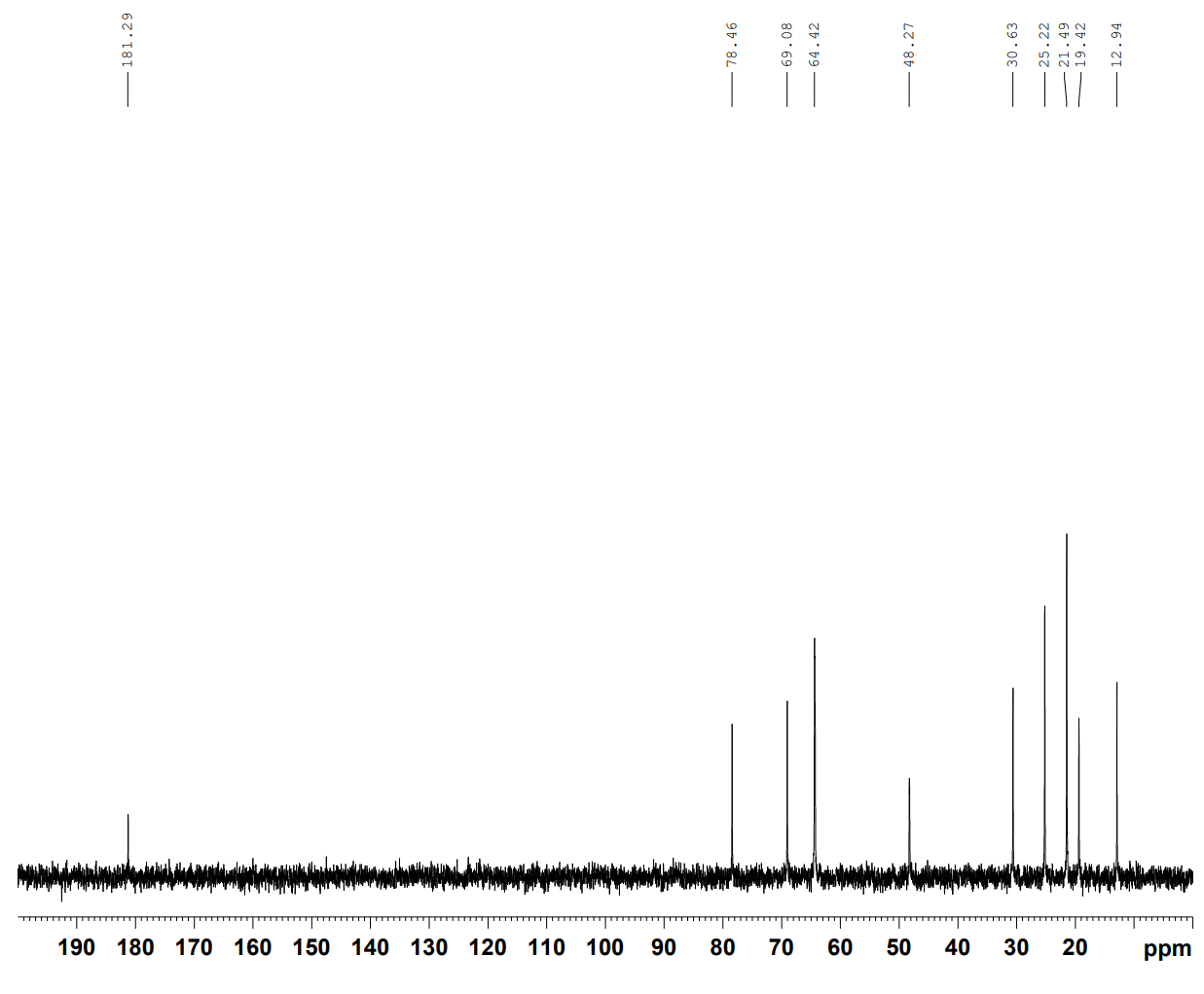
**

**Figure S14.** ^13^C spectrum of [BMPyrr][HFuA] in D_2_O.

**
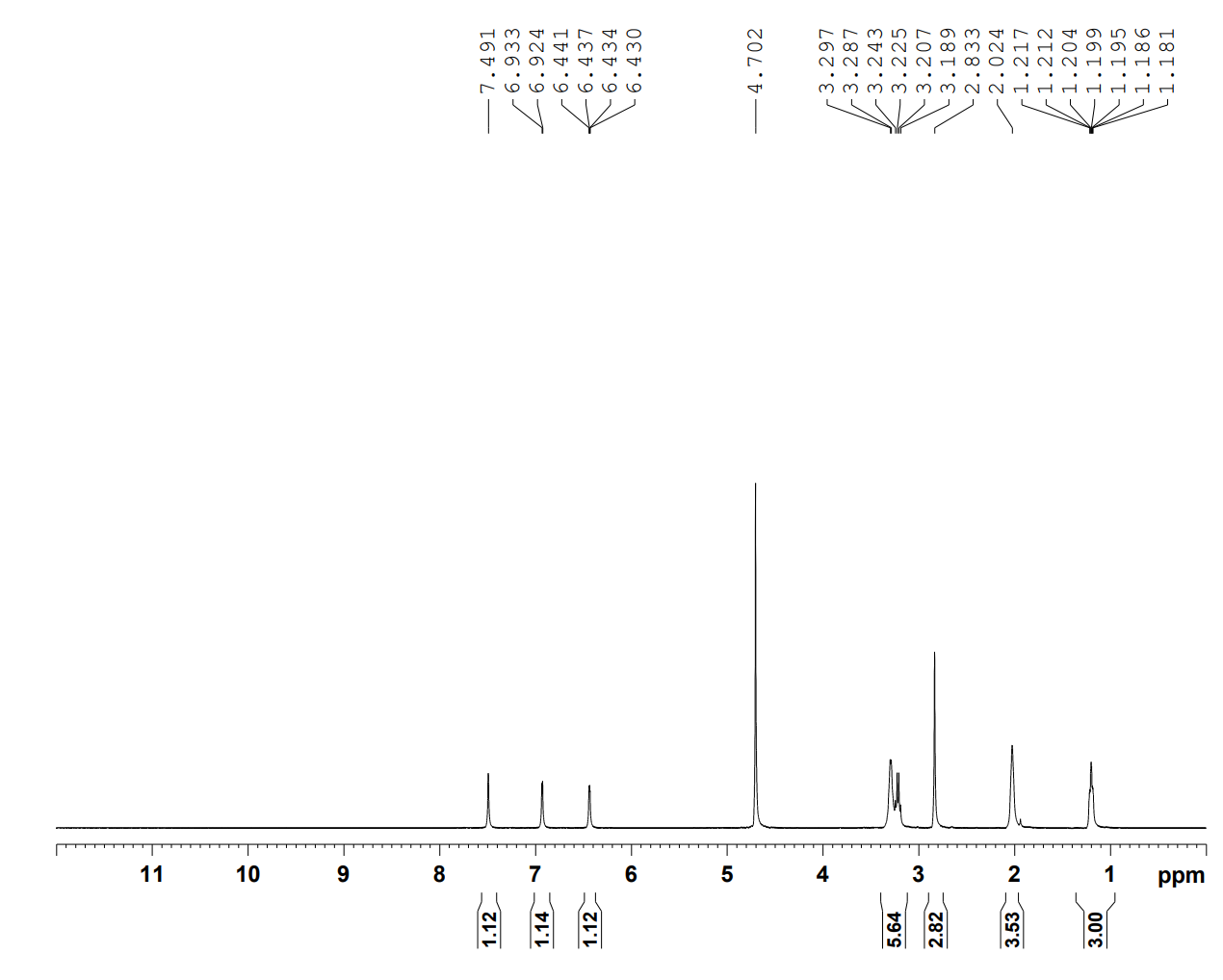
**

**Figure S15.** ^1^H spectrum of [EMPyrr][FuA] in D_2_O.

**
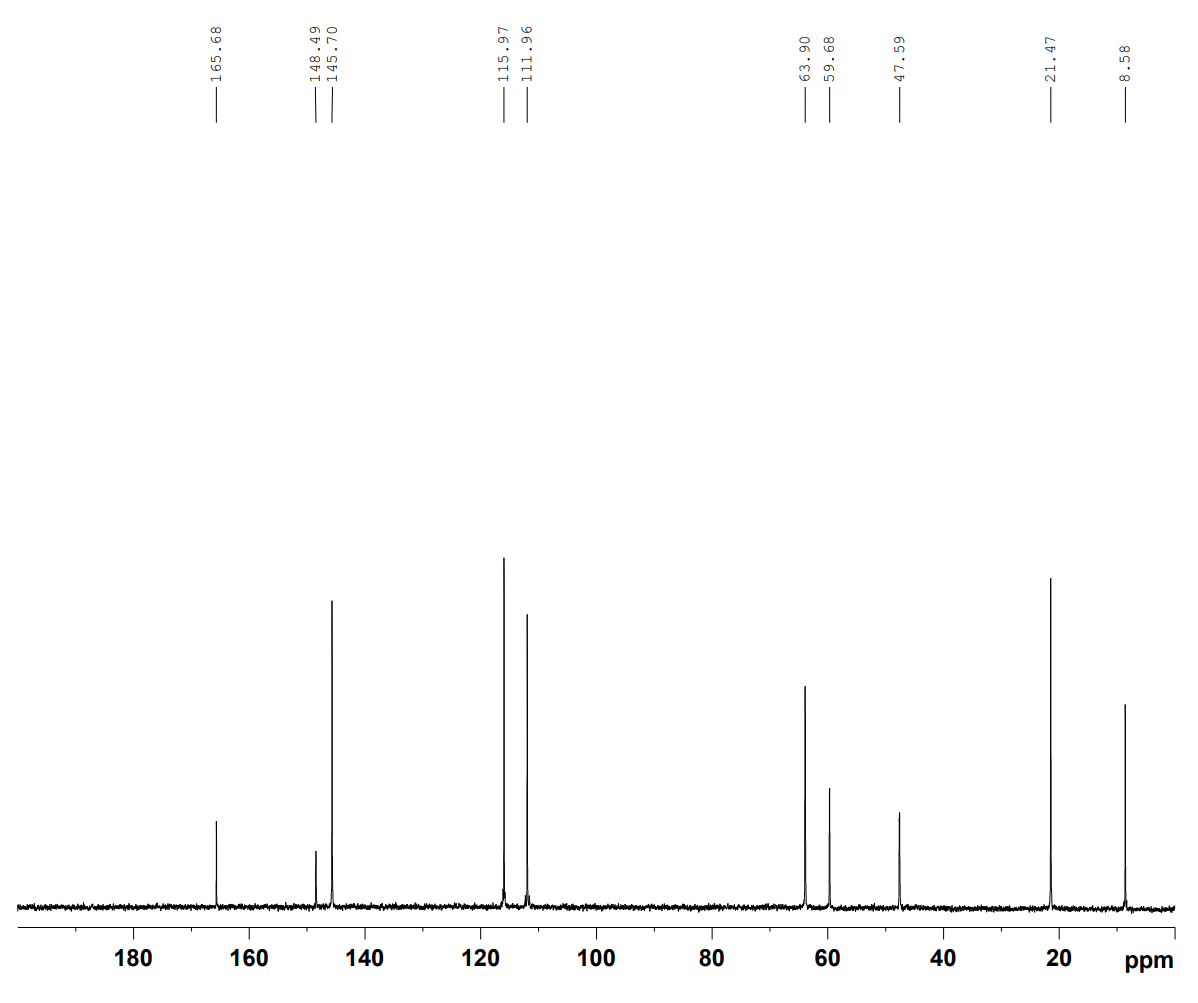
**

**Figure S16.** ^13^C spectrum of [EMPyrr][FuA] in D_2_O.

**
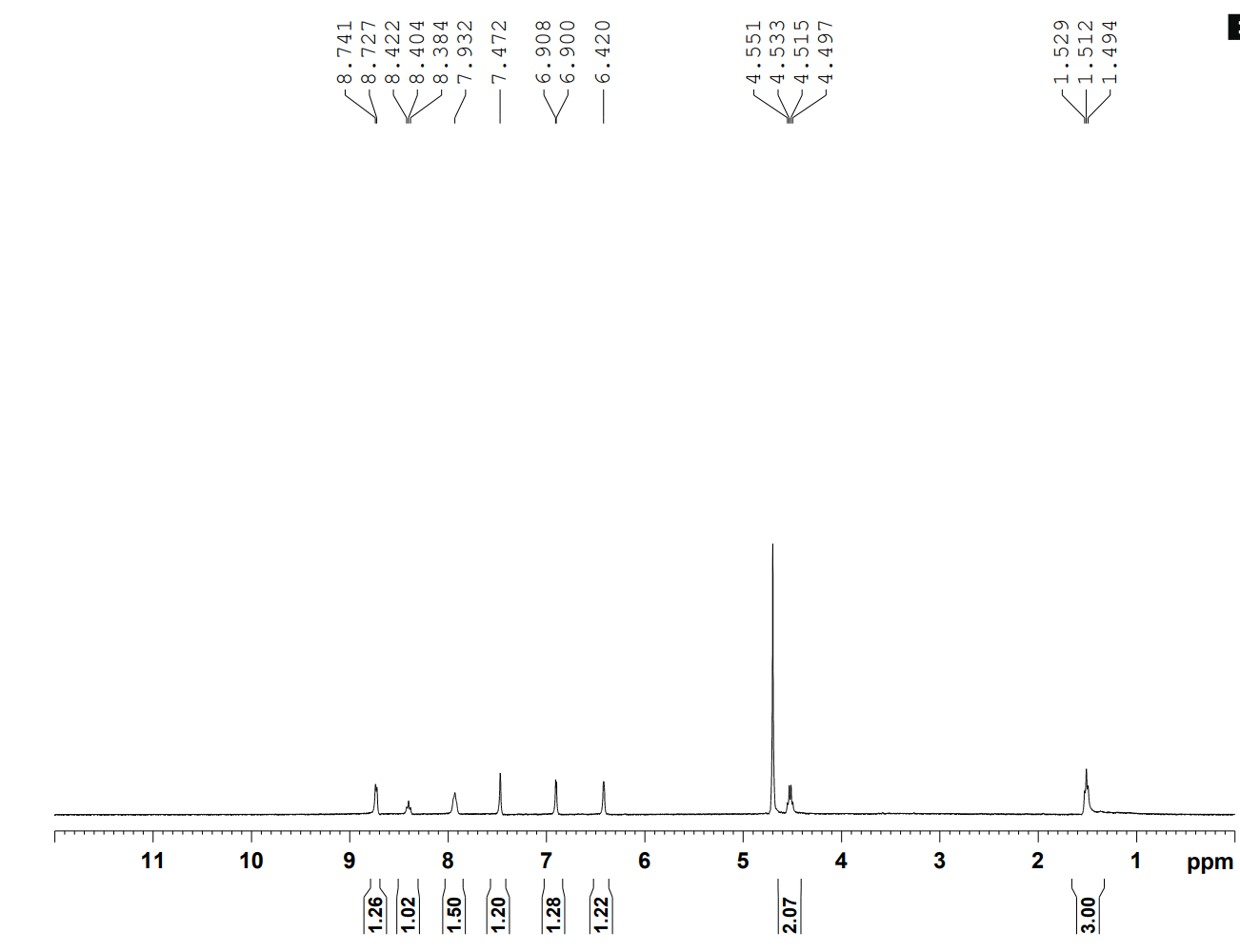
**

**Figure S17.** ^1^H spectrum of [EPy][FuA] in D_2_O.

**
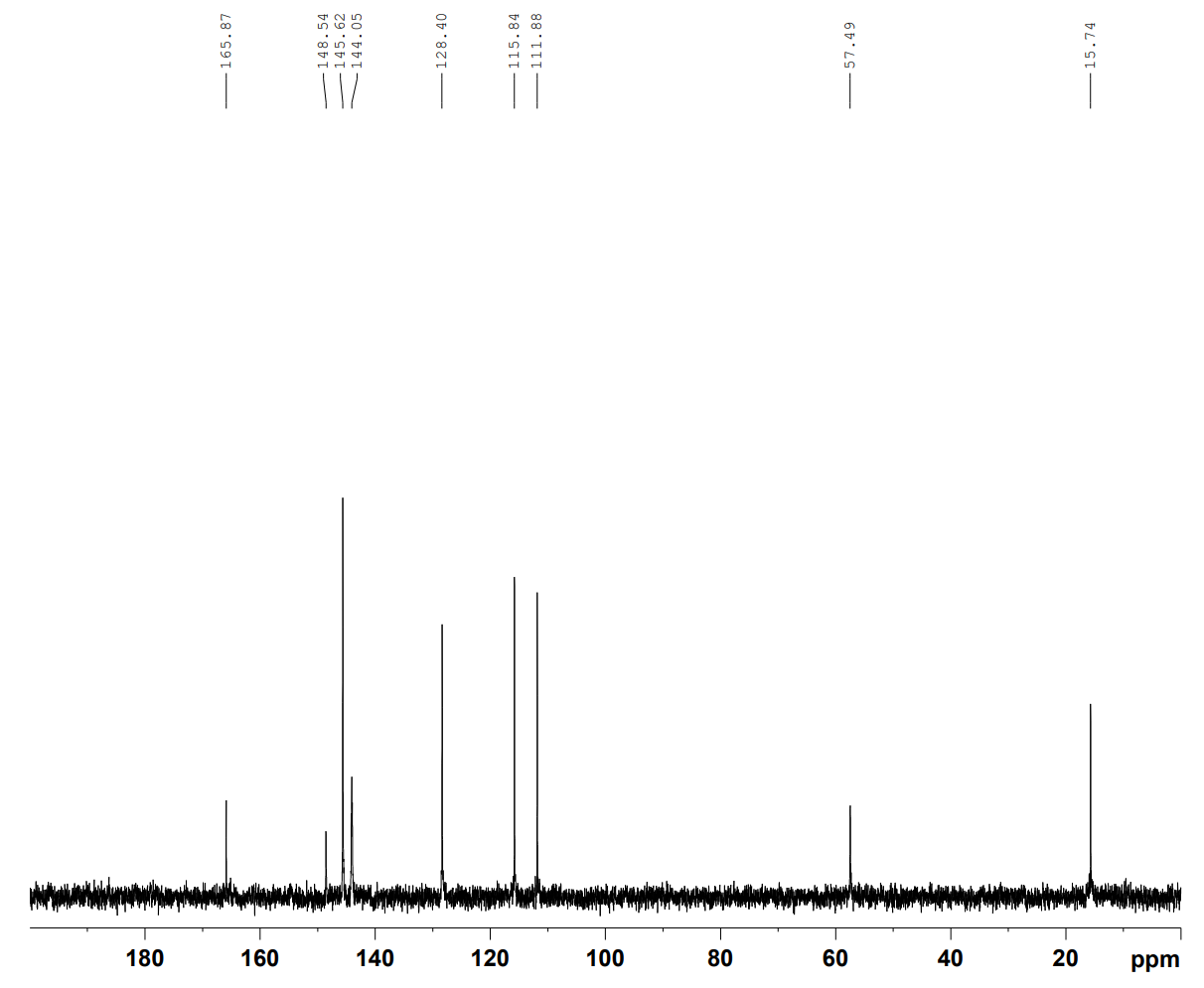
**

**Figure S18.** ^13^C spectrum of [EPy][FuA] in D_2_O.


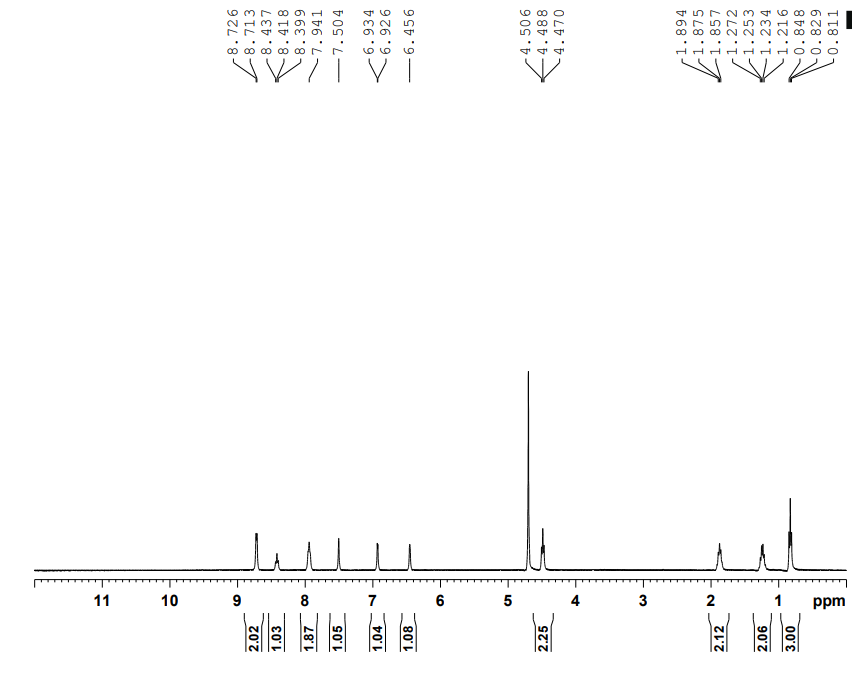


**Figure S19.** ^1^H spectrum of [BPy][FuA] in D_2_O.

**
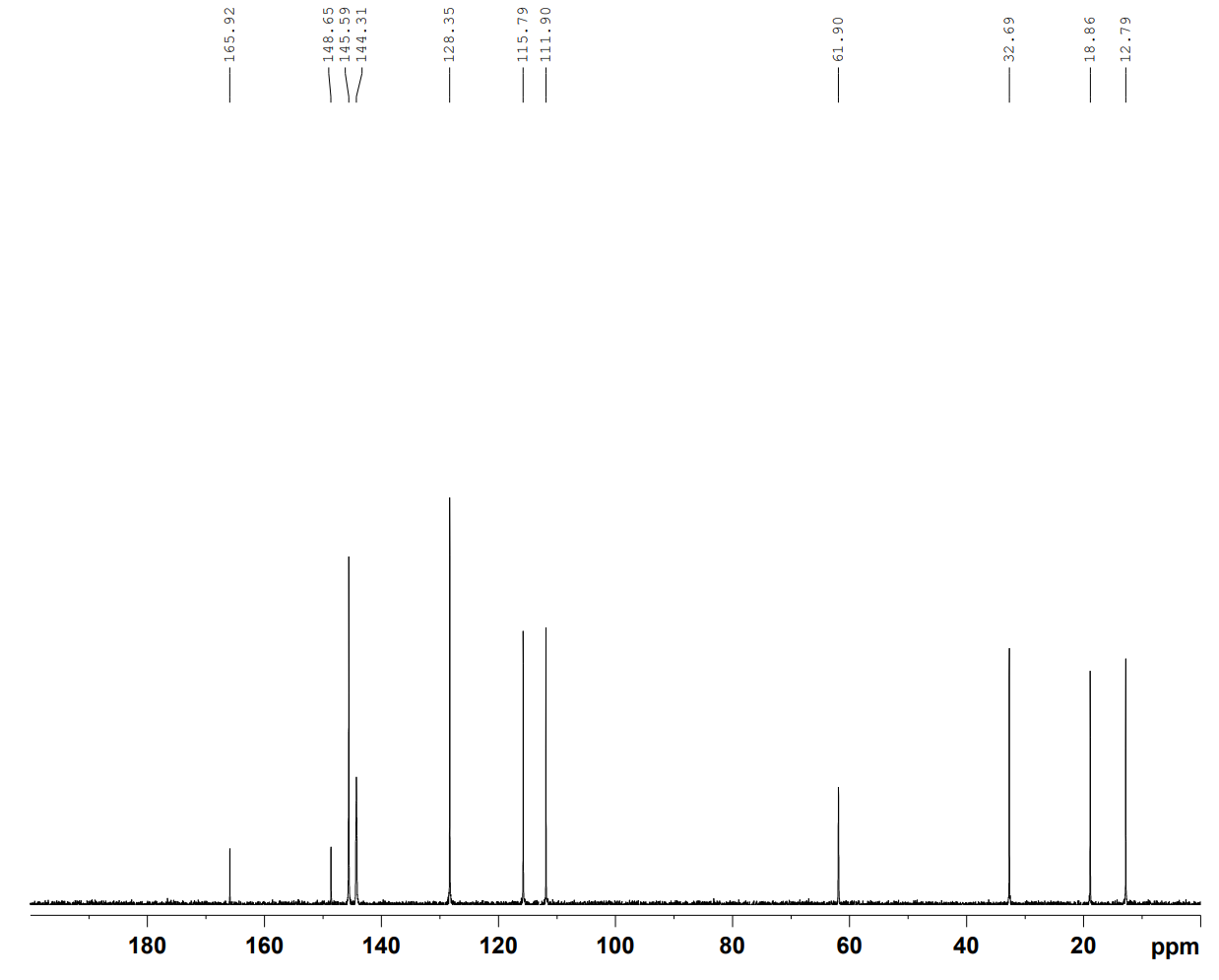
**

**Figure S20.** ^13^C spectrum of [BPy][FuA] in D_2_O.

**
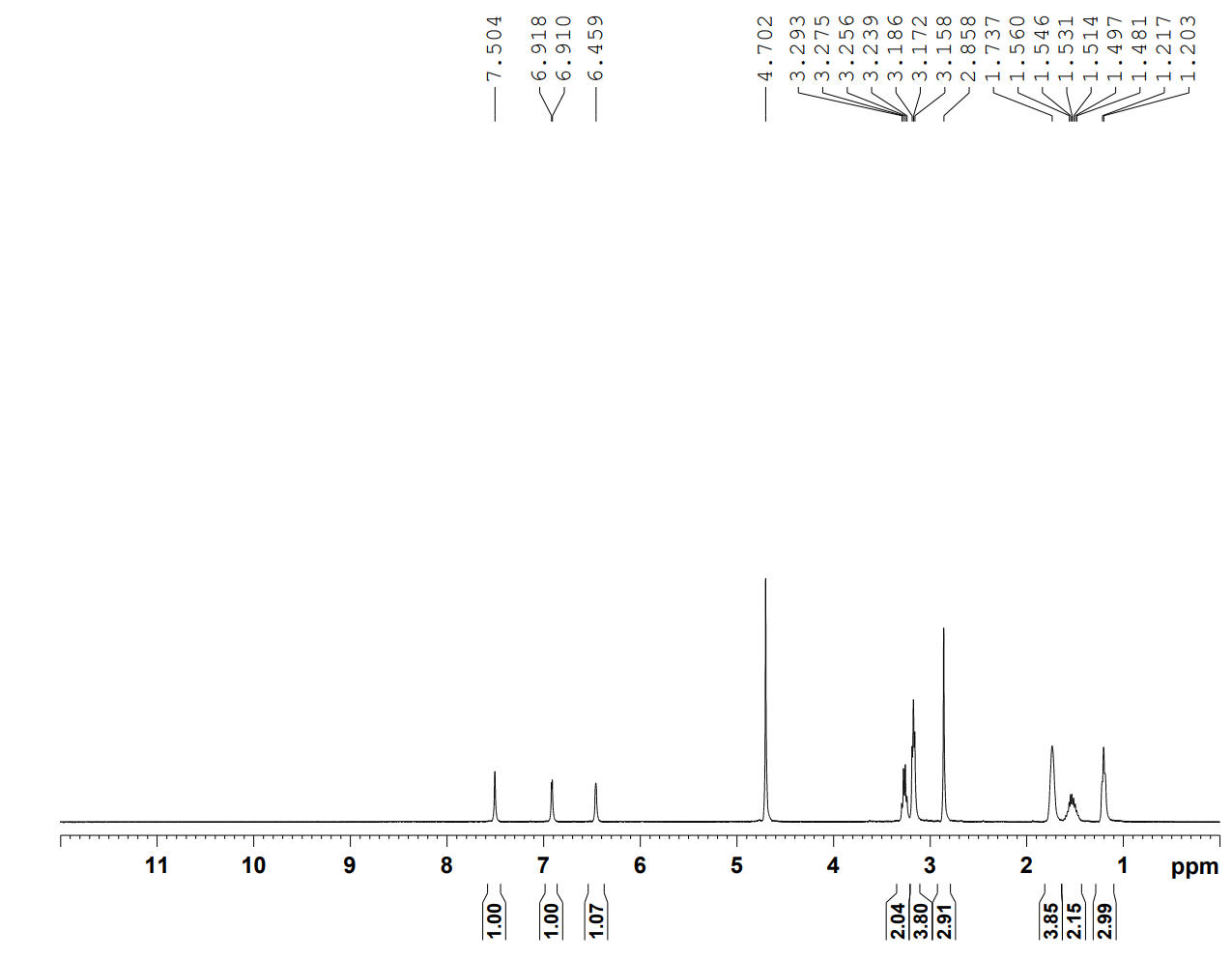
**

**Figure S21.** ^1^H spectrum of [EMPip][FuA] in D_2_O.

**
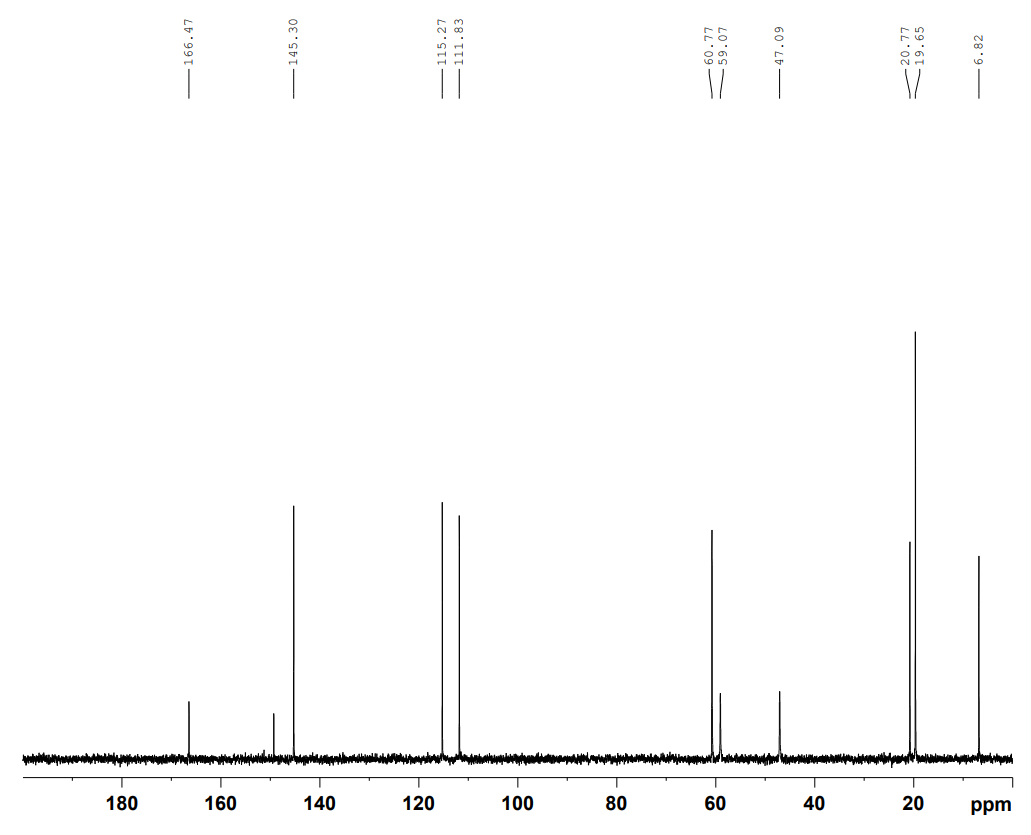
**

**Figure S22.** ^13^C spectrum of [EMPip][FuA] in D_2_O.

**
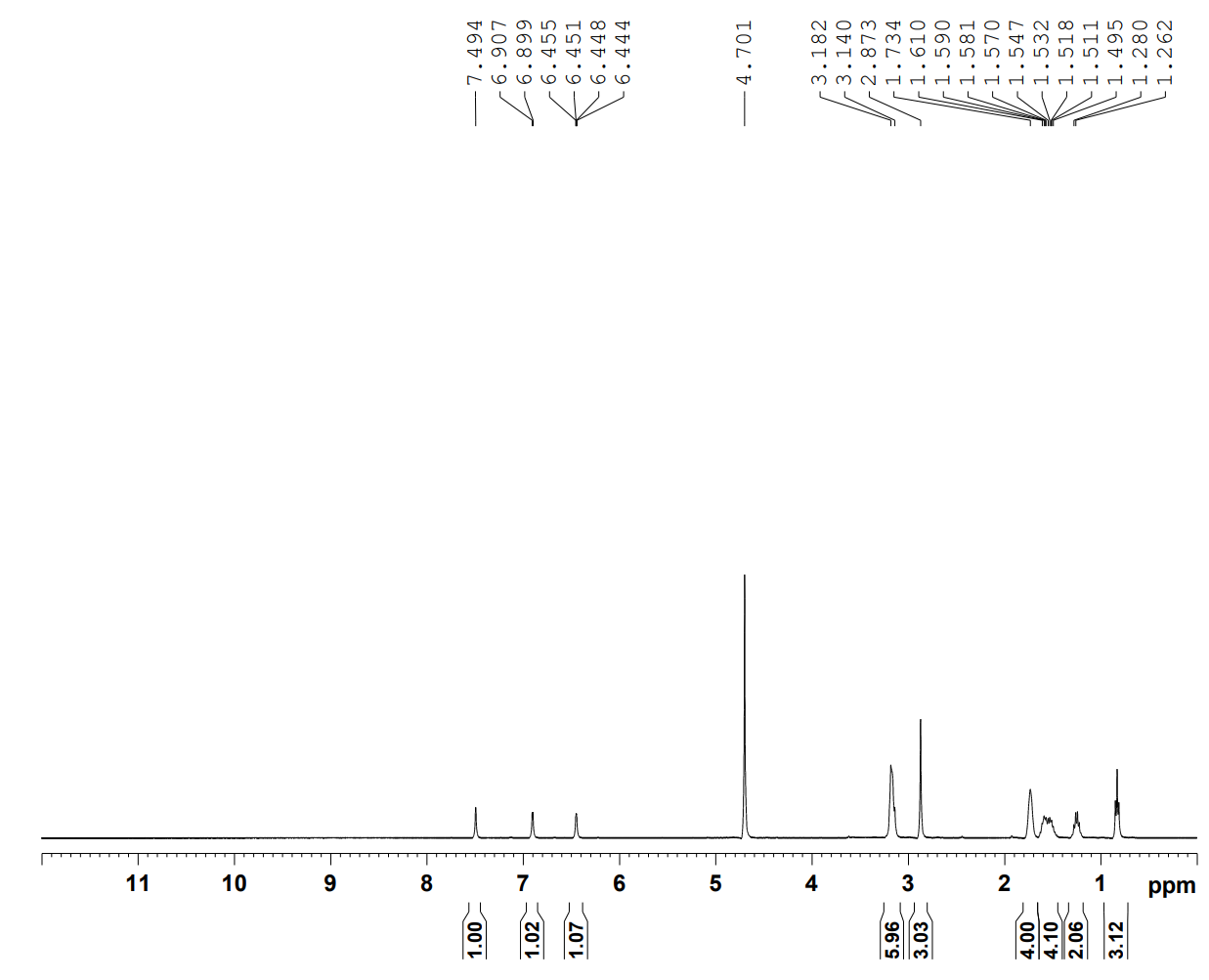
**

**Figure S23.** ^1^H spectrum of [BMPip][FuA] in D_2_O.

**
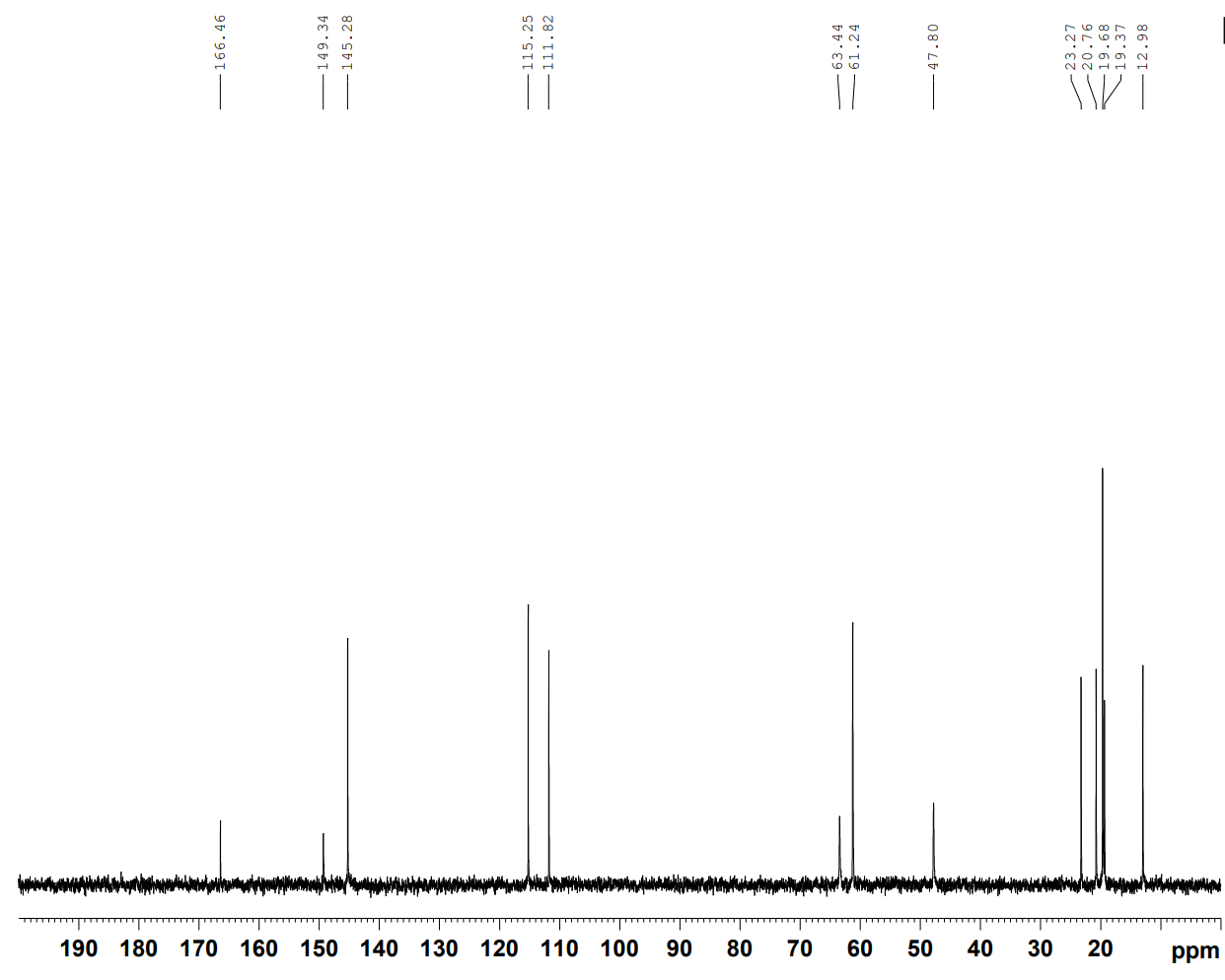
**

**Figure S24.** ^13^C spectrum of [BMPip][FuA] in D_2_O.

**
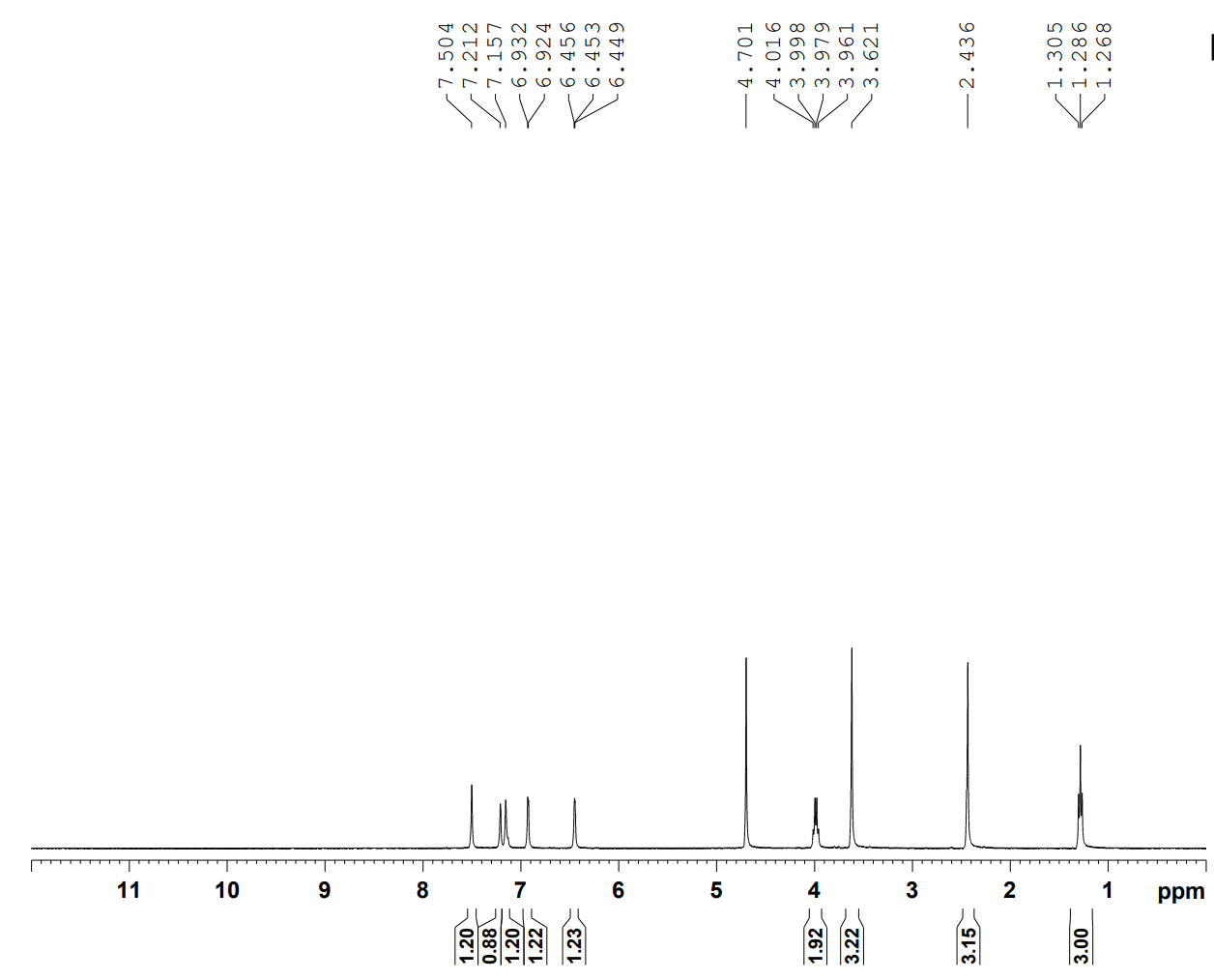
**

**Figure S25.** ^1^H spectrum of [EMMIm][FuA] in D_2_O.

**
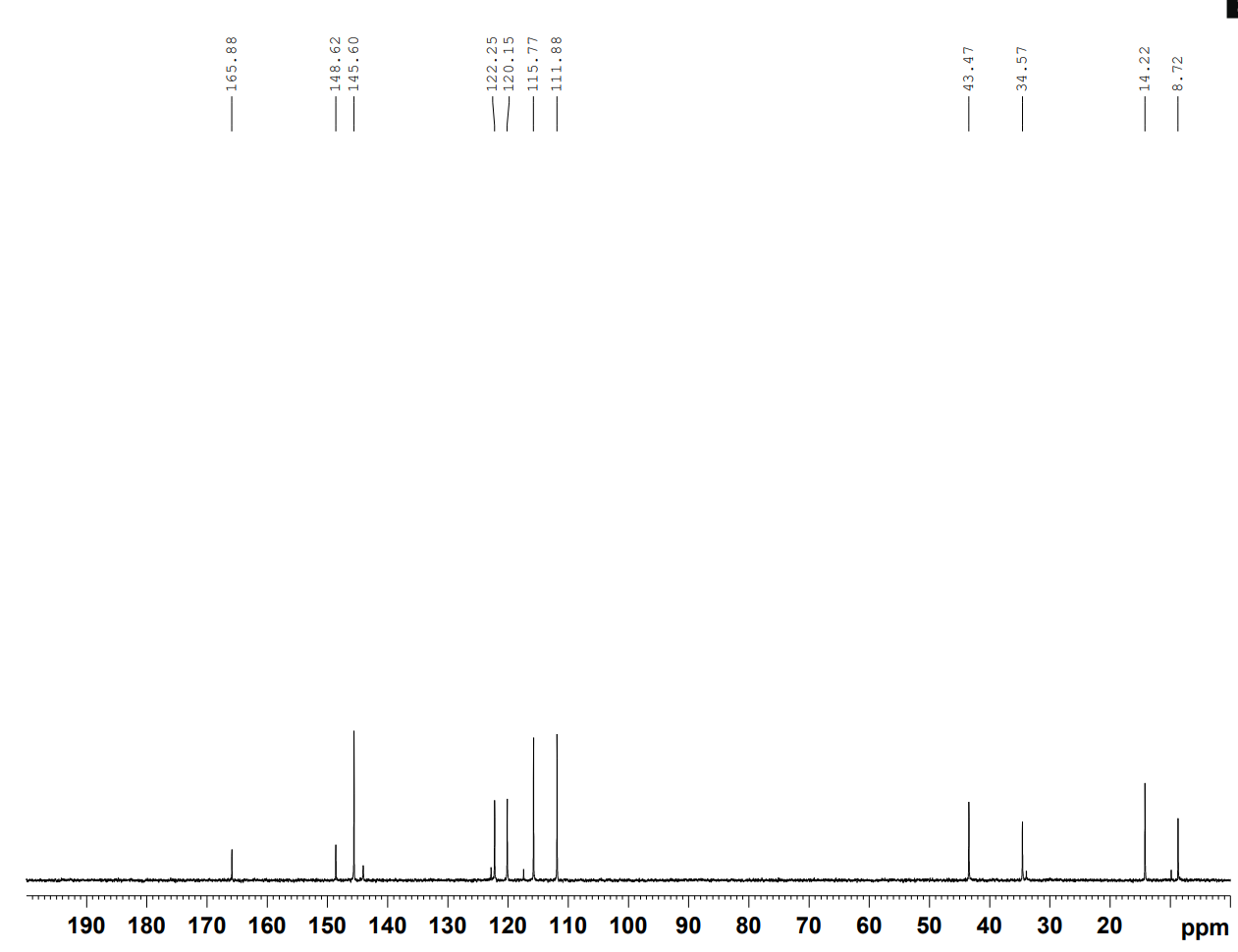
**

**Figure S26.** ^13^C spectrum of [EMMIm][FuA] in D_2_O.

**
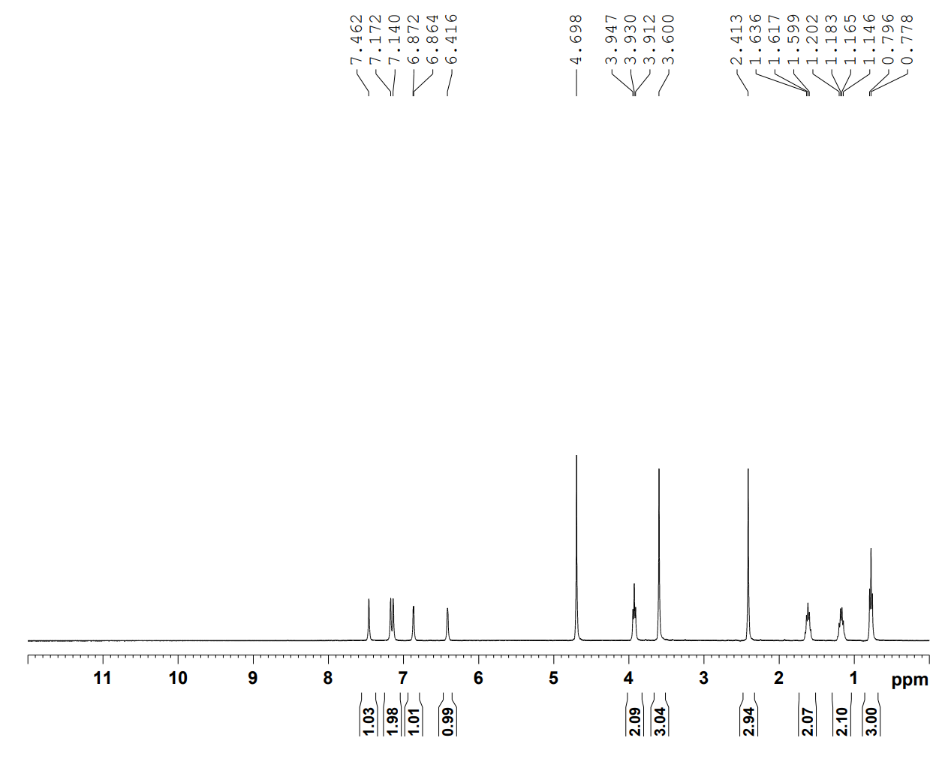
**

**Figure S27.** ^1^H spectrum of [BMMIm][FuA] in D_2_O.

**
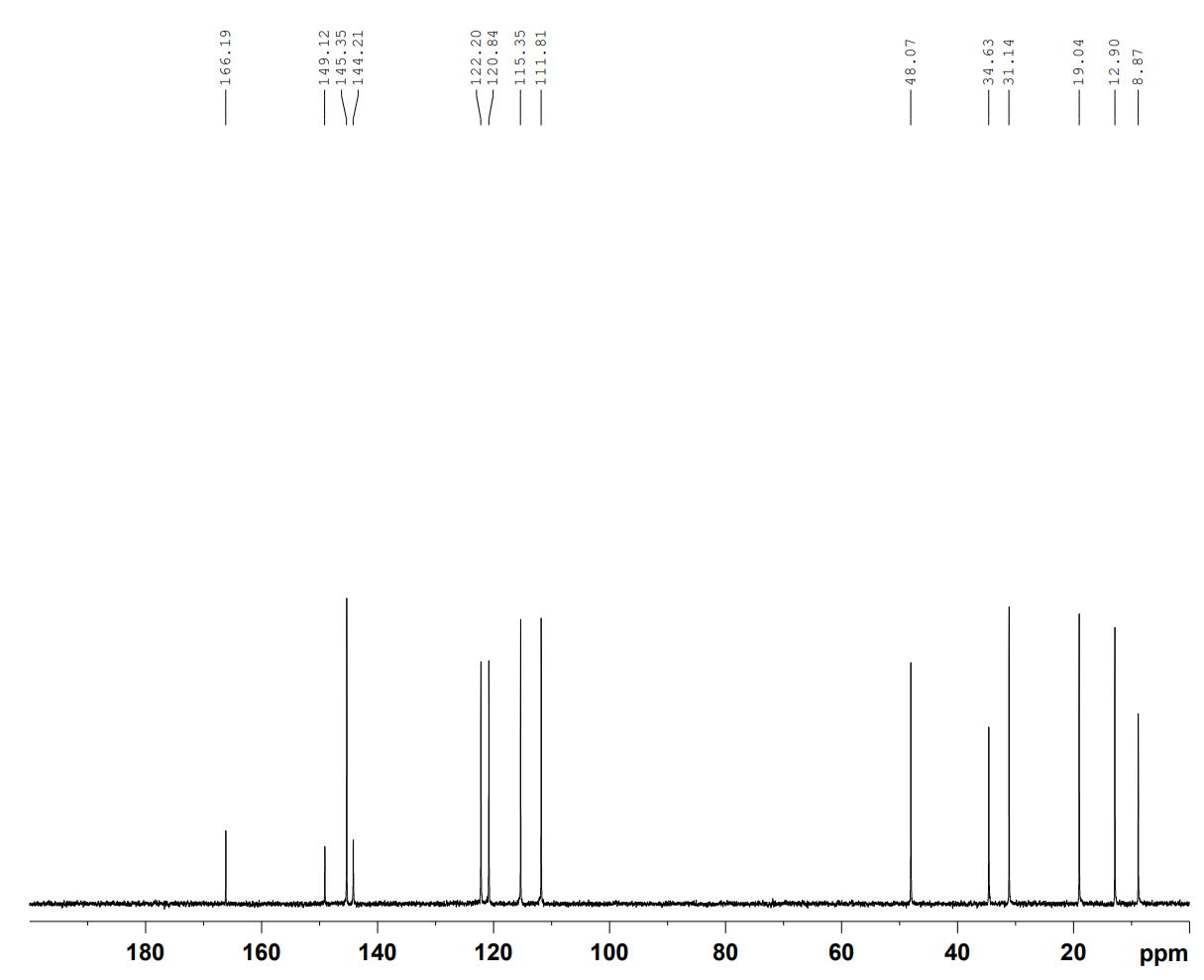
**

**Figure S28.** ^13^C spectrum of [BMMIm][FuA] in D_2_O.

**
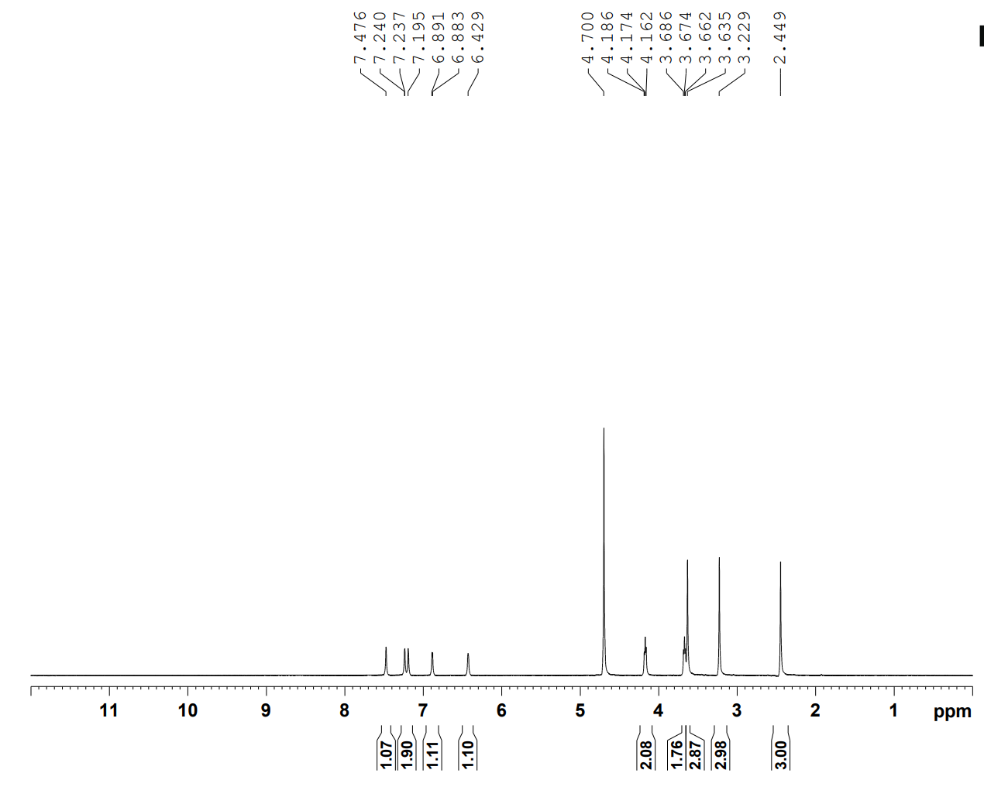
**

**Figure S29.** ^1^H spectrum of [BOMMIm][FuA] in D_2_O.

**
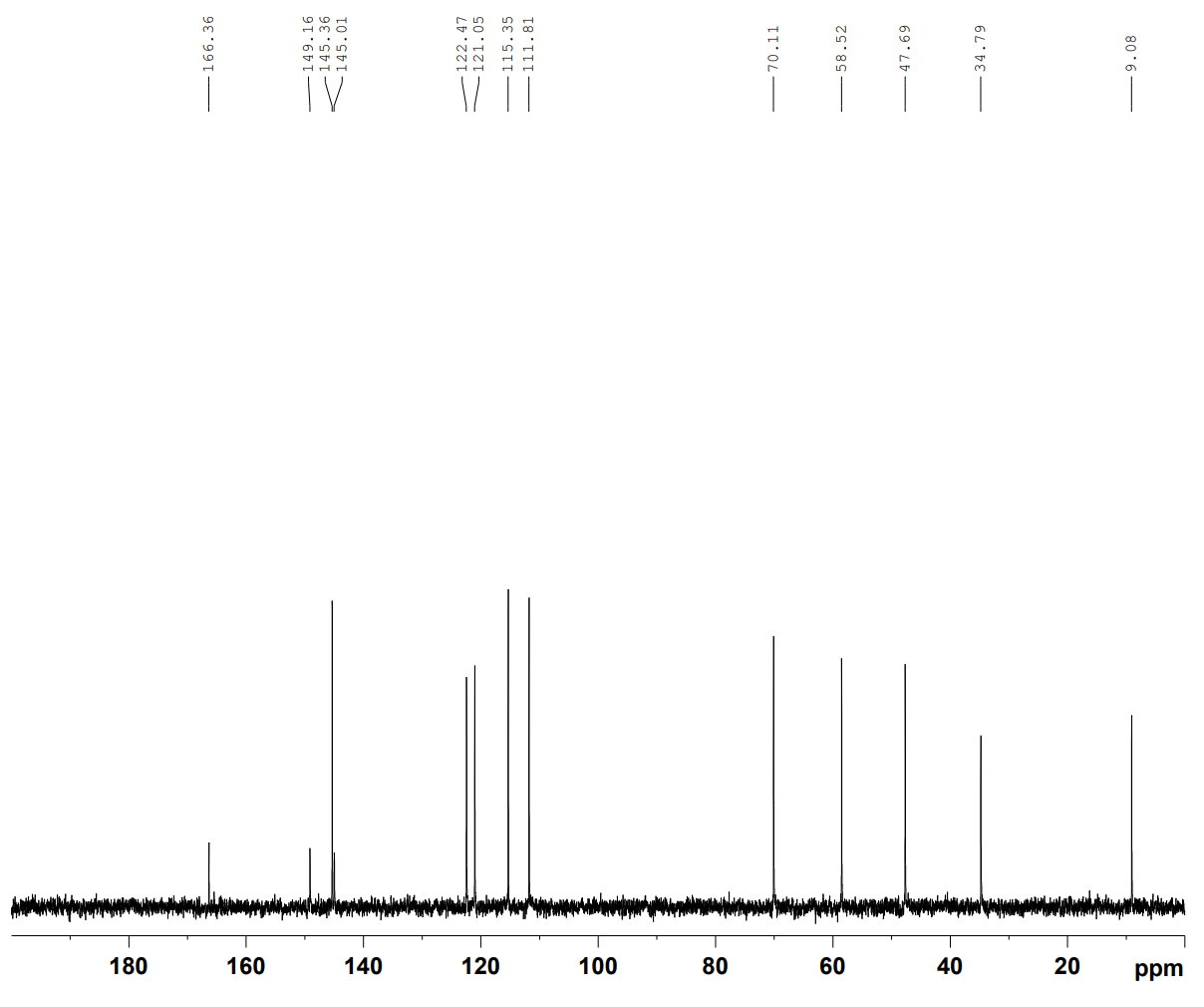
**

**Figure S30.** ^13^C spectrum of [BOMMIm][FuA] in D_2_O.

**Figure S31**. Nyquist plots of the (a) neat ILs at 20 ^o^C, and (b) [BMPyrr][FuA] as a function of temperature.

**Figure S32**. CV curves using different scan rates at 30 °C for the supercapacitors utilizing (a) [EPy][FuA], and (b) [EMPip][FuA] as electrolytes.

**Figure S33**. CV plots at 60 °C for (a, and c) [EPy][FuA] and (b, and d) [EMPip][FuA] at variable voltages. Nyquist plots of (e) [EPy][FuA] and (f) [EMPip][FuA] electrolytes.

**Figure S34**. Specific capacitance of the supercapacitors as a function of temperature and scan rates for (a) [EPy][FuA] and (b) [EMPip][FuA] electrolytes.

**Figure S35**. Coulombic efficiency of the supercapacitors with [EPy][FuA] and [EMPip][FuA] IL as electrolytes.

**Table S1.** VFT equation parameters of the ionic conductivity data for the synthesized ILs.

| IL | σ_0_, (mS cm^−1^) | Correlation coefficients *(R^2^ values)* | *B* (K) | *T*_0_ (K) | *E*_σ_ (kJ mol^−1^) |
| --- | --- | --- | --- | --- | --- |
| [BMPyrr][FuA] | 1.62 | 0.99978 | 952.64 | 185.38 | 7.96 |
| [BMPyrr][HFuA] | 1.32 | 0.99982 | 935.58 | 176.13 | 7.78 |
| [EMPyrr][FuA] | 1.17 | 0.99969 | 826.49 | 184.39 | 6.87 |
| [EPy][FuA] | 0.75 | 0.99942 | 582.45 | 198.54 | 4.84 |
| [BPy][FuA] | 0.38 | 0.99926 | 634.22 | 204 | 5.27 |
| [EMPip][FuA] | 1.71 | 0.99966 | 970.38 | 193.53 | 8.06 |
| [BMPip][FuA] | 1.51 | 0.99792 | 1098.64 | 190.60 | 9.13 |
| [EMMIm][FuA] | 0.23 | 0.99958 | 500.65 | 219.80 | 4.16 |
| [BOMMIm][FuA] | 0.88 | 0.99951 | 738.47 | 205.54 | 6.139 |

**Table S2.** VFT equation parameters of the ion diffusion data for the synthesized ILs.

| Ion | *D*_0_ (m^2^ s^−1^) | *B* (K) | *T*_0_ (K) | *E*_σ_ (kJ mol^−1^) |
| --- | --- | --- | --- | --- |
| [BMPyrr][FuA]-Anion | 1.09 $\times$ 10^-7^ | 2840 | 82 | 23.61 |
| [BMPyrr][FuA]-Cation | 3.02 $\times$ 10^-8^ | 1320 | 162 | 10.97 |
| [BMPyrr][HFuA] -Anion | 3.25 $\times$ 10^-9^ | 275 | 258 | 2.28 |
| [BMPyrr][HFuA]-Cation | 1.44 $\times$ 10^-8^ | 1154 | 155 | 9.59 |
| [EMPyrr][FuA] -Anion | 1.42 $\times$ 10^-6^ | 2866 | 82 | 23.82 |
| [EMPyrr][FuA] -Cation | 1.52 $\times$ 10^-6^ | 2998 | 66 | 24.92 |
| [EPy][FuA] -Anion | 1.03 $\times$ 10^-7^ | 1954 | 103 | 16.25 |
| [EPy][FuA] -Cation | 2.25 $\times$ 10^-8^ | 1103 | 170 | 9.17 |
| [BPy][FuA] -Anion | 7.25$\times$ 10^-8^ | 1733 | 140 | 14.40 |
| [BPy][FuA] -Cation | 8.13$\times$ 10^-8^ | 1647 | 147 | 13.69 |
| [EMPip][FuA] -Anion | 1.19$\times$ 10^-8^ | 944 | 200 | 7.84 |
| [EMPip][FuA] -Cation | 8.83$\times$ 10^-9^ | 879 | 202 | 7.30 |
| [BMPip][FuA] -Anion | 1.04 $\times$ 10^-6^ | 2704 | 104 | 22.48 |
| [BMPip][FuA] -Cation | 1.63 $\times$ 10^-6^ | 2932 | 94 | 24.37 |
| [EMMIm][FuA] -Anion | 4.5$\times$ 10^-8^ | 1425 | 166 | 11.84 |
| [EMMIm][FuA] -Cation | 1.32 $\times$ 10^-7^ | 1688 | 151 | 14.03 |
| [BMMIm][FuA] -Anion | 1.54 $\times$ 10^-8^ | 1044 | 194 | 8.67 |
| [BMMIm][FuA] -Cation | 2.30 $\times$ 10^-8^ | 1178 | 182 | 9.79 |
| [BOMMIm][FuA] -Anion | 2.72 $\times$ 10^-9^ | 981 | 184 | 8.15 |
| [BOMMIm][FuA] -Cation | 1.09 $\times$ 10^-6^ | 766 | 194 | 6.36 |

**Table S3.** Specific capacitance using different current densities at 60 °C for [EPy][FuA]-IL and [EMPip][FuA]-IL-based supercapacitors.

| Current density  (A g^−1^) | Specific capacitance  for [EPy][FuA]-SC  (F g^−1^) | Specific capacitance  for [EMPip][FuA]-SC  (F g^−1^) |
| --- | --- | --- |
| 0.2 | 99 | 70 |
| 0.4 | 92 | 53 |
| 0.5 | 89 | 29 |

**Table S4.** The EIS data-based resistance values at 60 °C for supercapacitors with [EPy][FuA] and [EMPip][FuA] electrolytes.

| Electrolyte | | Internal resistance (R_s_) in Ω | Charge transfer resistance (R_ct_)  in Ω | Total resistance (R_ov_) in Ω |
| --- | --- | --- | --- | --- |
| [EPy][FuA] | Before stability | 7 | 70 | 77 |
|  | After stability | 15 | 135 | 150 |
| [EMPip][FuA] | Before stability | 16 | 101 | 116 |
|  | After stability | 62 | 168 | 230 |

**Table S5.** Energy and power densities using different current densities at 60 °C for [EPy][FuA] and [EMPip][FuA]-IL-based supercapacitors.

| Electrolyte | Current density  (A g^−1^) | Energy density  (Wh kg^−1^) | Power density  (W kg^−1^) |
| --- | --- | --- | --- |
| [EPy][FuA] | 0.2 | 56 | 410 |
|  | 0.4 | 52 | 790 |
|  | 0.5 | 48 | 930 |
| [EMPip][FuA] | 0.2 | 36 | 360 |
|  | 0.4 | 28 | 750 |
|  | 0.5 | 14 | 870 |

**Table S6.** Energy densities for some reported IL-based supercapacitors.

| IL | Capacitance (Fg^−1^) | Cycle life | Power density (kW Kg^−1^) | Energy density  (Wh kg^−1^) | Electrode material | Ref. |
| --- | --- | --- | --- | --- | --- | --- |
| [EMIm][BF_4_]  [EMIm][DCA]  [EMIm][TFSI]  [BMPyrr][TFSI]  [BMPyrr][DCA] | 36  55  (From CV, 5mV s^˗1^)  30  31  58 | Not reported | 12.1  17.1  9.9  5.2  17.5 | 61  48  51  59  88 | Graphene nanosheets (GNSs) | 1 |
| [BMIm][PF_6_] | 158 (From GCD, current density: 0.2 A g^˗1^) | 3000 cycles | Not reported | 71 | Reduced graphene oxide (RGO) | 2 |
| [PMPyrr][TFSI] | 71.5 (From GCD, current density: 0.2 A g^˗1^) | 2000 cycles | 2.1 | 40 | Poly(ionic liquid)-modified RGO electrode (PIL:RGO) | 3 |

**References**

(1) Huang, P.-L.; Luo, X.-F.; Peng, Y.-Y.; Pu, N.-W.; Ger, M.-D.; Yang, C.-H.; Wu, T.-Y.; Chang, J.-K. Ionic Liquid Electrolytes with Various Constituent Ions for Graphene-Based Supercapacitors. *Electrochim. Acta* **2015**, *161*, 371–377.

(2) Chen, Y.; Zhang, X.; Zhang, D.; Yu, P.; Ma, Y. High Performance Supercapacitors Based on Reduced Graphene Oxide in Aqueous and Ionic Liquid Electrolytes. *Carbon N Y* **2011**, *49* (2), 573–580.

(3) Trigueiro, J. P. C.; Lavall, R. L.; Silva, G. G. Supercapacitors Based on Modified Graphene Electrodes with Poly(Ionic Liquid). *J. Power Sources* **2014**, *256*, 264–273.
